# Proteome-Wide Monitoring of Drug Action in Living Cells Using a Novel Label-Free Solvent-Based Shift Assay

**DOI:** 10.1016/j.mcpro.2025.101444

**Published:** 2025-11-05

**Authors:** Dominik Steinbrunn, Catalina Cepeleaga, Alexander Betz, Gözde Kibar, Melanie Holzner, Stefan K. Maier, Christin Zasada, Götz Hagemann, Stephan A. Sieber, Hannes Hahne

**Affiliations:** 1 OmicScouts GmbH, Lise-Meitner-Straße 30, Freising, Germany; 2 Department of Bioscience, TUM School of Natural Sciences, Technical University of Munich, Center for Functional Protein Assemblies (CPA), Garching bei München, Germany

**Keywords:** label-free drug target identification, intracellular target engagement, kinase inhibitor, HDAC inhibitor, molecular glue, PROTAC, CETSA

## Abstract

Biophysical proteomics assays allow for proteome-wide, label-free monitoring of ligand-induced changes in protein structure and stability, offering insights into protein-ligand interactions and modulation of biophysical properties of cellular proteins. These assays exploit the principle that compound-induced alterations in structure or stability of proteins can be detected through changes in their susceptibility to denaturation. Here, we introduce solvent proteome profiling in cells (SPICE), which employs solvent-based denaturation of proteins under otherwise physiological conditions in intact cells. We characterized solvent-induced denaturation of proteins inside cells as distinct from that in cell extracts and validated SPICE by detecting known drug-target interactions for multiple compound classes. Our results indicate that SPICE, unlike experiments in cell extracts, also detects secondary compound-induced effects such as target profiles of drug metabolites, modulation of protein-protein interactions, and downstream signaling events. We further demonstrate complementarity of SPICE and cellular thermal shift assay, which both robustly detect the designated targets of well-characterized drugs and individually provide biologically meaningful and interpretable results. Finally, we show that SPICE can detect covalent drug-targets, compound-induced target-destabilization and stabilization of degrader drug targets despite their concurrent degradation.

## Highlights

• Organic solvents allow for protein denaturation in live cells.
• SPICE allows for drug target identification in live cells.
• SPICE allows to infer biological downstream effects of inhibition of a drugs target.
• SPICE is complementary to CETSA.
• Modified format detects degrader target engagement despite concurrent degradation.

## Graphical Abstract

**Figure:**
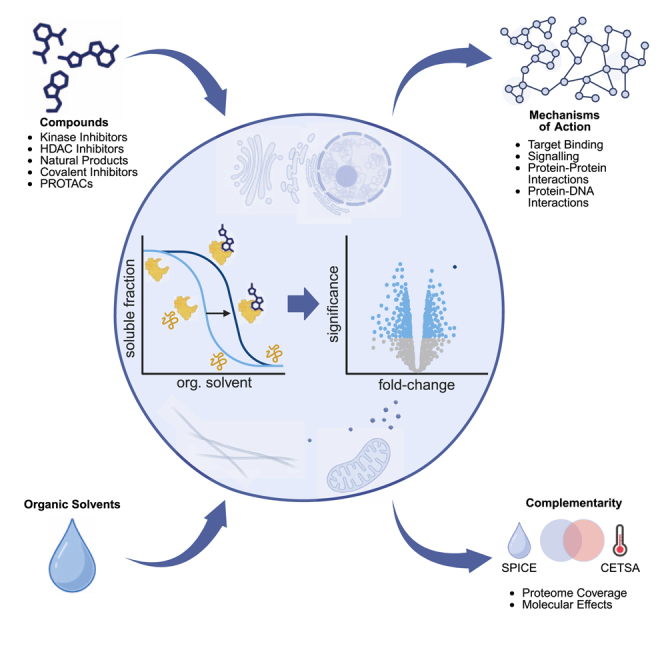

## In Brief

A method for solvent proteome profiling in cells has been developed. Protein denaturation by organic solvents in live cells was characterized and intracellular target engagement was detected for multiple compound classes. In contrast to lysate-based experiments, the method allowed to infer biological downstream effects of drug target inhibition. Denaturation profiling, target identification, and mechanism-of-action deconvolution provided complementary insights to the cellular thermal shift assay. Modification of the method enabled detection of intracellular target engagement despite concurrent degradation of target proteins.

Detecting interactions between a ligand and cellular proteins is crucial during drug discovery and development, as it facilitates drug-target identification and mechanism of action ((MoA), deconvolution, as well as identification of off-targets and potential liabilities (1). Proteomics using liquid chromatography coupled to tandem mass spectrometry (LC-MS/MS) enables the identification of such effects on a proteome-wide scale, and several LC-MS/MS-based methods have been developed to identify protein-ligand interactions across the entire proteome (2). Although affinity- and activity-based enrichment approaches have been successfully employed to identify proteome-wide target engagement for multiple small compounds (3), they rely on chemical modification of either the ligand or the protein, which might not be possible in some cases, and can lead to altered activity and specificity of the compound or the target protein (4, 5).

In contrast, modification-free approaches enable detection of drug-protein interactions free from these limitations (6) by detecting changes in biophysical properties of proteins interacting with a ligand: In drug affinity responsive target stability assays (DARTS), pulsed proteolysis assays and limited proteolysis assays (LiP-MS), interaction of a ligand with its target proteins is detected by an increased resistance to proteolysis (7, 8, 9). Chemical denaturant and protein precipitation assays, as well as stability of proteins from rates of oxidation assays detect compound-protein interactions by increased stability of targets in protein unfolding agents like urea or guanidinium chloride (10, 11), salts (12) or low pH (13). The most extensively employed methods are thermal proteome profiling (TPP) or cellular thermal shift assay (CETSA), which identify a ligand’s protein targets through detection of increased thermal stability upon protein-ligand interaction (14, 15, 16). Unlike most other label-free target engagement assays, TPP has not only been applied to cellular extracts, but also to live human cells, tissues, and blood (14, 15, 16, 17). Applications of TPP have demonstrated the ability to detect interactomes of metabolites (18) and enzyme substrates (19), protein-protein interactions and protein complex dynamics (20, 21, 22), as well as functionally distinct proteoforms (23).

Although CETSA has been proven useful in those cases, multiple studies have demonstrated the benefit of combining different (label-free) drug target identification methods to confidently identify ligand interactors and obtain a comprehensive picture of the relevant target space (13, 24, 25, 26, 27). One such alternative approach is solvent-induced protein precipitation, where protein-ligand interactions are identified by increased resistance against denaturation by organic solvents (28). The original method has been advanced through application of tandem mass tag (TMT)-based multiplexing as solvent-induced protein profiling (SIPP) and through an area-under-the-denaturation-curve approach as solvent-proteome integral solubility alteration (PISA) (27, 29, 30). Complementarity between TPP and SIPP for drug target identification in cellular extracts has been demonstrated in a few cases (27, 30).

TPP applied on live cells offers an advantage over lysate-based methods by detecting perturbed thermal stability of proteins directly in their native context (14, 17), rather than in cell extracts (15) or on purified proteins (31), which cannot fully reflect an intracellular environment (32). SIPP, in contrast, has been exclusively applied to cell extracts and is consequently limited in detecting biological effects of compounds beyond direct drug-target binding. We therefore have adapted the workflow of SIPP to enable modification-free drug target identification experiments with live cells under physiological conditions, calling it solvent proteome profiling in cells (SPICE). First, we characterized solvent-induced protein denaturation in live cells. We then demonstrate that SPICE can identify ligand induced secondary effects such as target engagement by a drug’s metabolic product or changes in protein-protein interactions and cellular signaling. Comparison to CETSA proved complementary coverage of assayable proteins, as well as largely nonoverlapping sets of proteins with compound-induced stability changes, which are individually biological meaningful and interpretable, but together provide a more comprehensive view on the stability-modulated proteome upon drug treatment.

## Experimental Procedures

### Cell Culture

Cells were obtained from American Type Culture Collection (ATCC) and cultured in the respective growth medium (Pan-Biotech), supplemented with 10% fetal bovine serum (Pan-Biotech). For experiments on lysates, cells were harvested, washed twice with Dulbecco’s phosphate-buffered saline (DPBS, Pan-Biotech) and stored as pellets at −80 °C until further use.

### Solvent-Induced Proteome Profiling

SIPP experiments were performed as described previously (27, 28). Proteins were extracted from frozen cell pellets by addition of lysis buffer (DPBS, 0.4% IGEPAL-630, 1X Halt protease, and phosphatase inhibitor cocktail (Thermo Fisher Scientific)) and three freeze-thaw cycles, using liquid nitrogen. Lysates were cleared by centrifugation for 30 min at 4 °C and 19,000g. The protein concentration of the soluble fraction was determined by a bicinchoninic acid assay (Thermo Fisher Scientific) and diluted to 4 μg/μl using lysis buffer. For treatments, dimethyl sulfoxide (DMSO) or compounds in DMSO were added 1:100 to a final concentration of 1% DMSO and incubated on a head-over-end shaker for 20 min at room temperature. Each sample was divided into 10 aliquots and exposed to increasing concentrations of AEA (acetone:ethanol:acetic acid, 50:50:0.1) to reach a final concentration of 2 μg/μl protein and final AEA concentrations from 0 to 27% (v/v) (0, 3, 6, 9, 12, 15, 18, 21, 24, and 27%). All samples were then incubated at 37 °C and under vigorous shaking for 20 min. Precipitated proteins were separated by centrifugation at 4 °C and 4000g for 45 min. Equal amounts of the soluble fraction were removed and used for LC-MS/MS sample preparation.

### Solvent Proteome Profiling in Cells

Previously published protocols for SIPP (27, 28, 30) were adapted to allow for SPICE. Cells were cultured in the respective growth medium until a maximum density of 80%, harvested, and washed twice with DPBS. For treatment, the cells were resuspended in prewarmed medium containing a compound or DMSO in the desired final concentration, followed by incubation for 1 hour at 37 °C and gentle shaking to avoid settling of the cells. Both suspension and adherent cells were kept in suspension during treatment. Treated cells were pelleted, washed with DPBS (Pan-Biotech) and resuspended in resuspension buffer (DPBS, 1X protease and phosphatase inhibitor cocktail (Thermo Fisher Scientific)). Cell suspensions were divided into 10 aliquots of one million cells per aliquot and exposed to increasing concentrations of AEA from 0 to 20% (v/v) (0, 8, 10, 11, 12, 13, 14, 15, 16, 18, and 20%). All samples were then incubated at 37 °C and vigorous shaking for 20 min. For complete lysis, IGEPAL-630 was added to a final concentration of 0.4% and cells were lysed by three freeze-thaw cycles using liquid nitrogen. Precipitated proteins and cell debris were separated by centrifugation at 4 °C and 4000g for 45 min. Equal amounts of the soluble fraction were removed and used for LC-MS/MS sample preparation.

### SIPP or SPICE in a Compressed Format

For SIPP or SPICE in a compressed format, treated samples were exposed to AEA concentrations from 8 to 22% (8, 10, 12, 14, 16, 18, 20, and 22%) and equal amounts of the soluble fractions were pooled into a single sample per denaturation curve before LC-MS/MS sample preparation. For parallel analysis of abundance changes, an aliquot of treated cells was processed the same way as SPICE samples, except for denaturation.

### Cellular Thermal Shift Assay

The cellular thermal shift assay was conducted as described previously (15, 33). Cells were cultured in the respective growth medium until a maximum density of 80%, harvested, and washed twice with DPBS. For treatment, the cells were resuspended in pre-warmed medium containing a compound or DMSO in the desired final concentration, followed by incubation for 1 hour at 37 °C and gentle shaking to avoid settling of the cells. Both suspension and adherent cells were kept in suspension during treatment. Treated cells were pelleted, washed with DPBS (Pan-Biotech) and resuspended in resuspension buffer (DPBS, 1X protease and phosphatase inhibitor cocktail (Thermo Fisher Scientific)). Cell suspensions were divided into eleven aliquots of one million cells per aliquot and exposed to increasing temperatures (37.0, 40.6, 42.9, 45.5, 50.8, 55.2, 57.2, 60.3, 65.3, 70.6, and 76.9 °C). For complete lysis, IGEPAL-630 was added to a final concentration of 0.4% and cells were lysed by three freeze-thaw cycles using liquid nitrogen. Precipitated proteins and cell debris were separated by centrifugation at 4 °C and 4000g for 45 min. Equal amounts of the soluble fraction were removed and used for LC-MS/MS sample preparation.

### PISA

For PISA, samples were generated as described for CETSA, but treated samples were exposed to temperatures from 51 to 64 °C (50.9, 53.2, 54.7, 56.4, 58.1, 59.8, 61.4, and 64.0 °C) and equal amounts of the soluble fractions were pooled into a single sample per denaturation curve as described before (29), followed by LC-MS/MS sample preparation.

### LC-MS/MS Sample Preparation

Soluble protein fractions were dried by vacuum centrifugation and resuspended in resuspension buffer (50 mM TEAB, 5% SDS, pH 8.5) before sample preparation based on protein aggregation capture (34). Peptides were eluted from the beads using 50 mM Hepes pH 8.5 containing trypsin (Promega, V5517) at a ratio of 1:25 (trypsin:protein). Samples were digested overnight at 37 °C under vigorous shaking. Eluted peptides were collected, and beads were washed with water to retrieve the remaining peptides. Both fractions were pooled and dried by vacuum centrifugation. Peptides were resuspended in water and TMT-labeling with TMT 10plex or TMTpro 16/18plex reagents was performed as previously described (35). After confirmation of successful labeling (labeling efficiency >98%), excess TMT reagents were quenched by addition of hydroxylamine to a final concentration of 0.4% and incubation at room temperature for 15 min. Equal amounts of labeled peptides were pooled in 0.5% formic acid (FA) and desalted using a Sep-Pak Vac 50 mg tC18 cartridge (Waters) according to the manufacturer’s protocol. The peptides were eluted in 60% acetonitrile, 0.1% FA, and dried using a vacuum concentrator. Desalted peptides were chromatographically separated via basic reversed phase chromatography as previously described (36) into 24 fractions, dried down, and stored at −20 °C until further analysis. For samples that were measured in data-independent aquisition (DIA) mode (experiments with MZ1, JQ1, and AHPC), the digestion buffer was 100 mM ammonium-bicarbonate and peptides were desalted using C18-cartridges on an Agilent Bravo Liquid Handling Platform before drying and storage at −20 °C until further analysis.

### LC-MS/MS Data Acquisition

For LC-MS/MS measurement of TMT labeled samples, dried peptides were resuspended in 0.1% FA. Data were acquired on a Q-Exactive Plus mass spectrometer (Thermo Fisher Scientific) coupled to an UltiMate3000 RSLCnano system (Thermo Fisher Scientific). Peptides were collected on a trap column (75 μm × 2 cm, packed in-house with ReproSil-Pur 120 ODS-3 resin, Dr Maisch) and separated on an analytical column (75 μm × 55 cm, packed in-house with Reprosil-Gold 120 C18, 3 μm resin, Dr Maisch) using a 120 min gradient from 2% to 32% solvent B (0.1% FA, 5% DMSO in HPLC-MS grade acetonitrile) in solvent A (0.1% FA, 5% DMSO in HPLC-MS grade water) at 300 nl/min. For TMT analysis, MS1 data were collected at 70,000 resolution, AGC target of 3e6 and 25 ms maximum injection time. The top 20 peptides with charges between two and six were selected for MS2 acquisition with an isolation window of 1.2 m/z and a dynamic exclusion time of 35 s. MS2 data were collected at 35,000 resolution, ACG target of 2e6 and 54 ms maximum injection time.

For LC-MS/MS measurement of label-free samples (experiments with MZ1, JQ, and AHPC and concentration resolved experiments), dried peptides were resuspended in 0.1% FA. Data were collected on an Exploris 480 mass spectrometer (Thermo Fisher Scientific) coupled to a Vanquish Neo UHPLC system (Thermo Fisher Scientific). Peptides were collected on a trap column (Acclaim PepMap 100 with 3 μM particle size, 75 μm inner diameter and 150 mm length) and separated on an analytical column (75 μm × 55 cm, packed in-house with Reprosil-Gold 120 C18, 3 μm resin, Dr Maisch) using a 120 min gradient from 2% to 32% solvent B (0.1% FA, 5% DMSO in HPLC-MS grade acetonitrile) in solvent A (0.1% FA, 5% DMSO in HPLC-MS grade water) at 300 nl/min. For DIA analysis, Full scan spectra (m/z 400 − 1000) were recorded at a resolution of 60,000, AGC target of 3e6 and a maximum injection time of 20 ms. MS2 scans were recorded at 30,000 resolution utilizing 45 m/z-windows. HCD collision was set to 27%, AGC target to 3e6, and a maximum injection time set to automatic.

### Protein Identification and Quantification

For TMT data, MaxQuant Software (v2.0.3.1) and the Andromeda Search Engine were used for identification and quantification of peptides and proteins (37). Database search was against a human protein database from SwissProt (SP000005640, canonical + isoform, download 2022, 42,404 entries). Isotope impurities of the TMT lot were specified to allow MaxQuant the automated correction of TMT intensities. Methionine oxidation and acetylation of protein N termini were set as variable modifications. Carbamidomethylation of cysteines was set as fixed modification. Isotope impurities of the TMT lot were specified to allow for correction of TMT intensities. Trypsin/P was set as the proteolytic enzyme and the maximum number of missed cleavages was set to 2. Results were adjusted to 1% false discovery rate for peptide spectrum, protein, and site match, employing a target-decoy approach using reversed protein sequences. Furthermore, default settings for the matching tolerances were used, which are 4.5 ppm for precursors, 20 ppm for MS/MS (FTMS), and 0.5 Da for MS/MS (ITMS).

For DIA data, DIA-NN (v1.8.1) was used for identification and quantification of peptides and proteins (38). Database search was against a human protein database from SwissProt (SP000005640, canonical + isoform, download 2023, 42,454 entries). Deep learning-based features were switched on. Methionine oxidation, acetylation of protein N termini and N-terminal methionine excision were set as variable modifications. Carbamidomethylation of cysteines was set as fixed modification. Trypsin/P was set as the proteolytic enzyme and the maximum numbers of missed cleavages and variable modifications were set to 2. The default parameter 0.0 was used for mass and retention time correction, leading to DIA-NN performing automatic mass and retention time correction. Match between runs and Heuristic protein interference was allowed, and precursor false discovery rate was set to 1%.

### Quantitative MS Data Analysis

For analysis of complete denaturation curves, MaxQuant output ProteinGroups.txt was filtered for contaminants, reversed peptides, proteins only identified by site and proteins, and proteins that were identified by less than two peptides. Relative intensities were calculated by dividing each intensity by the average of the respective 0% AEA samples over all replicates. Relative intensities were normalized toward a reference denaturation curve based on median relative intensities per % AEA. Curves were filtered for R^2^ > 0.8 and a bottom plateau <0.3.

Analysis for experiments in a compressed format was conducted using R (39) and RStudio (40). MaxQuant ProteinGroups.txt was filtered for contaminants, reversed peptides, proteins only identified by site and proteins, ad proteins that were identified by less than two peptides. DIA-NN output tables were filtered for contaminants. Intensities were normalized by median centering between TMT channels. Significant changes between treatments were determined using the limma package (41) including Benjamini-Hochberg-correction of *p* values (42). For experiments comparing abundance and stability changes, the respective fold-changes from both experiments were divided by each other, before plotting the resulting values on a log_2_-scale.

### Clustering and Enrichment Analysis

Hierarchical clustering of denaturation curves was performed using the stats R package for Euclidean clustering. All enrichment and functional clustering analyses were performed with DAVID (43), using all proteins quantified in the respective dataset as background. *p* values were adjusted by Benjamini-Hochberg correction. Analysis, clustering, and creation of figures for networks of proteins significantly changed in abundance or stability were performed using the apps stringApp (v2.1.1) (44) and clusterMaker2 (v2.3.4) (45) in Cytoscape (v3.10.1) (46) at the default score cutoff of 0.4.

### Visualization

Plots were produced using the ggplot2 R package (47), Cytoscape (46) or BioRender (Created in BioRender (2024) BioRender.com/v44b609.)

### Experimental Design and Statistical Rationale

Denaturation curve experiments for comparison of protein denaturation in cells and lysate and for target identification of methotrexate (MTX) were performed with eleven AEA concentrations. Replicate aliquots of cells and lysates were jointly cultured and prepared but separately treated and exposed to organic solvents. Samples belonging to the same denaturation curve and replicate were prepared in parallel for MS-analysis until pooling after TMT-labeling. All samples from the same denaturation curve and replicate were labeled with the same TMT plex. The median of samples with 0% AEA was used as reference for calculating relative values within each condition.

SPICE and SIPP experiments in the compressed format and full proteome experiments were performed in triplicates. Cells for different replicates and conditions were cultured jointly but treated separately, including treatment with DMSO in triplicates as a control. TMT samples were labeled with the same TMT plex with resulting reporter intensities being used for quantification. Quantified intensities for TMT labeled samples and label-free measured samples were median-centered for normalization. Statistical analysis was performed with the limma R package (41).

## Results

### SPICE Allows for Intracellular Protein Denaturation

**Fig. 1:**
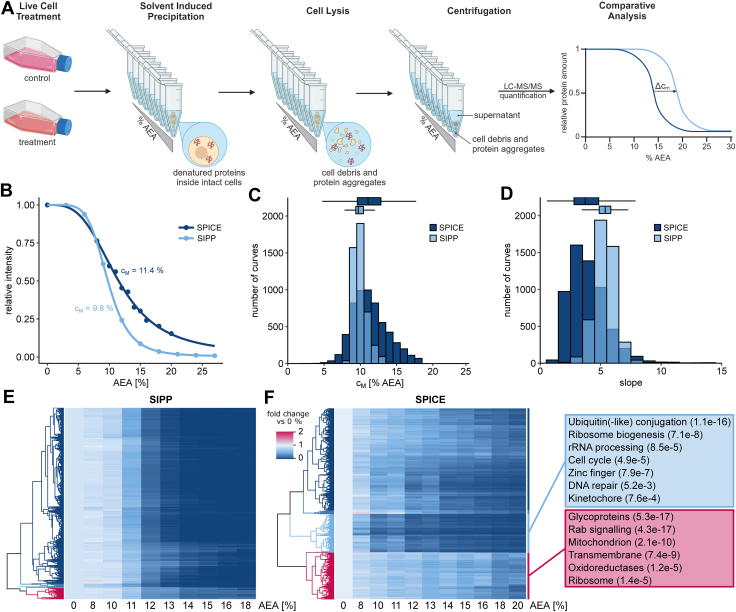
**Solvent proteome profiling in cells allows for intracellular protein denaturation**. *A*, schematic depiction of the SPICE workflow. *B*, median curves from denaturation experiments with cells (SPICE, *dark blue*, n = 1) and with lysate (SIPP, *light blue*, n = 2). *C* and *D*, histograms of c_M_-distributions and slope distributions from SPICE (*dark blue*) or SIPP (*light blue*) experiments. *E*, heatmap of denaturation curves from SIPP experiments. Intensities for each protein are shown relative to the intensity at 0% AEA. Proteins are ordered by hierarchical clustering. *F*, heatmap of denaturation curves from SPICE experiments. Intensities for each protein are shown relative to the intensity at 0% AEA. Proteins are ordered by hierarchical clustering. *Boxes* on the right indicate functional clusters of annotations from multiple databases including GO, KEGG, and UniProt, that are enriched in the respective hierarchical cluster. All proteins with curves passing the quality criteria were used as background. AEA, acetone:ethanol:acetic acid; SPICE, solvent proteome profiling in cells; SIPP, solvent-induced protein precipitation; GO, Gene Ontology; KEGG, Kyoto Encyclopedia of Genes and Genomes.

We developed a label-free biophysical proteomics method, termed SPICE, to enable analysis of protein-drug interactions in live cells based on SIPP (27, 28, 30) (Fig. 1*A*). For SPICE, live cells are treated with the desired ligand, and aliquots of the treated cells are then exposed to a gradient of the same organic solvent mixture that is used for SIPP (AEA 50:50:0.1 (v/v)). After denaturation, cells are lysed by the addition of 0.4% NP-40 and multiple freeze-thaw cycles. NP-40 solubilizes membrane proteins, without affecting ligand protein interactions (48). Protein precipitates and cell debris are removed by centrifugation and the soluble fraction of proteins in the supernatant is analyzed by quantitative proteomics.

To evaluate the effect of the solvent on the integrity of intact cells, we exposed K562 cells to AEA but omitted the cell lysis step and measured protein concentration in the supernatant after centrifugation. Without cell lysis, we measured less soluble protein in the supernatant than in the supernatant of cells that were exposed to AEA and additionally lysed (Supplemental Fig. S1*A*). This indicates that proteins are retained in intact cells during denaturation by AEA for most of the AEA concentrations used. At the strongest denaturing conditions, we observed partial lysis of cells by AEA, shown by increased protein amount in the supernatant. This is comparable to loss of membrane integrity at high temperatures during CETSA (15), and occurs mainly at AEA concentrations which are higher than the ones used in the following experiments. We hypothesized that AEA enters the cells during SPICE at concentrations which did not lyse the cells and gradually denatures proteins therein, manifesting in a distinct denaturation behavior compared to SIPP experiments in lysate.

To compare protein denaturation in cells to denaturation in lysate, we measured protein denaturation curves by exposing aliquots of live K562 cells and K562 cell extracts to increasing AEA concentrations using the SPICE or SIPP workflow. Soluble fractions were analyzed by quantitative TMT-based proteomics. Denaturation curves for each protein were modeled based on the measured protein intensities assuming two-state unfolding and filtered for sufficient quality using criteria commonly applied in TPP and SIPP studies (R^2^ > 0.8, bottom plateau <0.3) (16, 30). Median denaturation curves over all proteins for the two opposed conditions show distinct overall denaturation (Fig. 1*B*). The median melting concentration (c_M_) for SIPP is 9.8% AEA, consistent with previous reports (30), while the c_M_ for SPICE is 11.4% AEA. Furthermore, the median curve for SPICE possesses a shallower slope, while still reaching a bottom plateau below the commonly used filtering criterion for protein denaturation curves. Interestingly, c_M_-values from SPICE for the individual proteins are not uniformly shifted to higher AEA concentrations but exhibit a broader distribution than for denaturation in lysate (Fig. 1*C*). Likewise, the distribution of slopes in SPICE experiments is not only shifted to lower values but also has a broader distribution (Fig. 1*D*). Furthermore, heatmaps for all denaturation curves from SIPP and SPICE experiments confirm the less homogenous denaturation behavior for proteins in cells (Fig. 1, *E* and *F*).

We further characterized the solvent-induced protein denaturation profiles from SPICE by hierarchical clustering and enrichment analyses. Proteins in the cluster containing the most stable proteins show enrichment of terms related to glycosylation, Rab signaling, oxidoreductases, ribosomal, membrane, and mitochondrial proteins (Supplemental Fig. S1*B*). In the low stability cluster, terms related to conjugation to ubiquitin or similar proteins are enriched most significantly and for the highest number of proteins (Supplemental Fig. S1*C*). For mitochondrial and membrane proteins, high stability is consistent with observations from TPP studies (33) and can be rationalized with the molecular interactions of those protein classes. Glycosylation stabilizes proteins against thermal denaturation (49) and facilitates protein-protein interactions. Also, glycoproteins are mostly membrane associated and localized in the endoplasmic reticulum and organelles emerging thereof (50), which explains the high intracellular stability due to their protein-membrane interactions. Rab signaling proteins and oxidoreductases interact with metabolites in cells, which stabilize proteins (18, 19). These metabolites are diluted during cell lysis, likely resulting in the relative destabilization of Rab signaling proteins and others in lysate (Supplemental Fig. S1*D*). The high stability of ribosomal proteins in cells, and their relative stabilization compared to lysates, might be caused by intact intracellular ribosomal complexes, in which complex members stabilize each other (16). Notably, when contrasting denaturation of proteins of the two ribosomal subunits, there is a significant difference between the c_M_-values of proteins belonging to the small or large ribosomal subunit in lysate, but not in cells (Supplemental Fig. S1*E*). We assume this can be caused by ribosomal subunits which are separated upon lysis, but are still stable enough to be intact and therefore coaggregate in SIPP experiments, akin to previously observed thermal coaggregation of tightly interacting proteins (22, 51, 52). In contrast, intracellular ribosomal subunits seem to coaggregate as intact ribosomes. For membrane proteins, increased stability in SPICE experiments is expected, since they are known to be stabilized within intact membranes through protein-lipid interactions (53). Subsequently, 78 of the 166 mitochondrial proteins in this cluster are membrane proteins as well. Fifty-nine are proteins of the mitochondrial ribosome, which we expect to be stabilized for the same reasons as the cytosolic ribosome. The remaining 20 proteins located in the mitochondrial matrix, form an interesting group, which could be protected from denaturation by AEA during experiments in cells, if the solvents penetrate the mitochondrial membrane either slower or overall less efficiently than the plasma or other cellular membranes. Functional enrichment of proteins in the low stability cluster correlates with previously shown biophysical destabilization of proteins by ubiquitin(-like) modifications (54). Cell cycle proteins and proteins localized at the kinetochore are in the same cluster, potentially by localization of the related processes in the cytosol, which might be the cellular compartment which is most directly accessible for AEA. Furthermore, proteins related to ribosome biogenesis, rRNA processing, DNA repair, and zinc finger proteins are enriched in the cluster of early precipitating proteins. All these processes involve nucleic acid binding in the nucleus, which might already be interfered by low amounts of solvents and destabilize proteins akin to thermal destabilization of proteins by removal of metal ligands (55).

Taken together, these observations indicate that protein denaturation during SPICE is strongly influenced by the physiological local environment, arising from protein-specific localizations and biomolecular interactions. We therefore conclude that SPICE allows denaturation of proteins within intact cells and enables monitoring of compound induced localization- and interaction-dependent intracellular protein stability.

### SPICE Enables Target Identification Specific to Live Cell Treatments

**Fig. 2:**
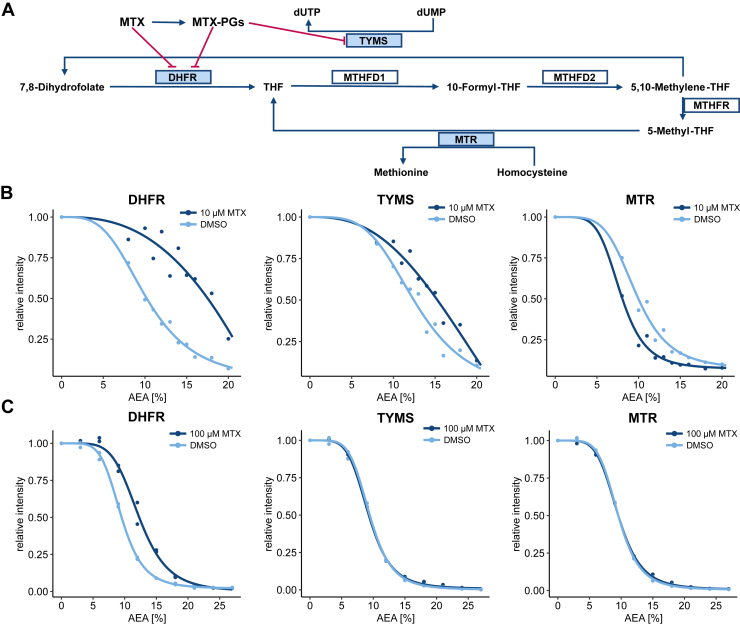
**SPICE can detect metabolization-dependent target engagement of MTX**. *A*, schematic depiction of the folate cycle and the role of methotrexate (MTX) and methotrexate-polyglutamates (MTX-PGs). *B*, K562 cells were treated with 10 μM MTX. Denaturation curves for the three known target proteins of MTX, DHFR, TYMS, and MTR are shown for treated (*dark blue*) or untreated (*light blue*) cells. *C*, K562 lysate was treated with 100 μM MTX. Denaturation curves for the three known target proteins of MTX, DHFR, TYMS, and MTR are shown for treated (*dark blue*) or untreated (*light blue*) lysate. DHFR, dihydrofolate reductase; TYMS, thymidylate synthase; SPICE, solvent proteome profiling in cells.

To assess whether SPICE allows detecting known ligand-protein interactions, which are specific to experiments with live cells, we contrasted SIPP and SPICE, using MTX as treatment. MTX it is a well characterized and specific ligand for dihydrofolate reductase (DHFR) (56), but is also known to undergo polyglutamylation in metabolically active cells, leading to an additional interaction with thymidylate synthase (TYMS) (Fig. 2*A*). K562 cells and lysates were treated with 10 μM or 100 μM MTX for 1 hour or 30 min respectively, subjected to either SPICE or SIPP, and analyzed by quantitative TMT-based proteomics (Supplemental Datasets S1 and S2). DHFR was stabilized in both experiments, whereas TYMS was only stabilized in SPICE experiments (Fig. 2, *B* and *C*). TYMS stabilization by MTX has previously been observed with CETSA (15) but not with SIPP (28), highlighting the capability of SPICE to detect drug metabolite interactions beyond the interactions detectable by SIPP. The relatively higher effect size on cells is likely due to the active import of MTX and retention of polyglutamylated MTX, resulting in an increased local concentration. In addition to DHFR and TYMS, methionine synthase (MTR), another protein within the folate cycle, was exclusively destabilized in the SPICE experiment. Inhibition of DHFR and TYMS decreases intracellular concentrations of several metabolites within the folate cycle, such as MTR’s substrate, 5-methyl-tetrahydrofolate (57); the reduced ligand interaction may lead to a relative destabilization of the protein, similar to destabilization observed for metal-binding proteins after removal of metal ligands (55).

### Protein Denaturation in SPICE Is Different and Complementary to CETSA

**Fig. 3:**
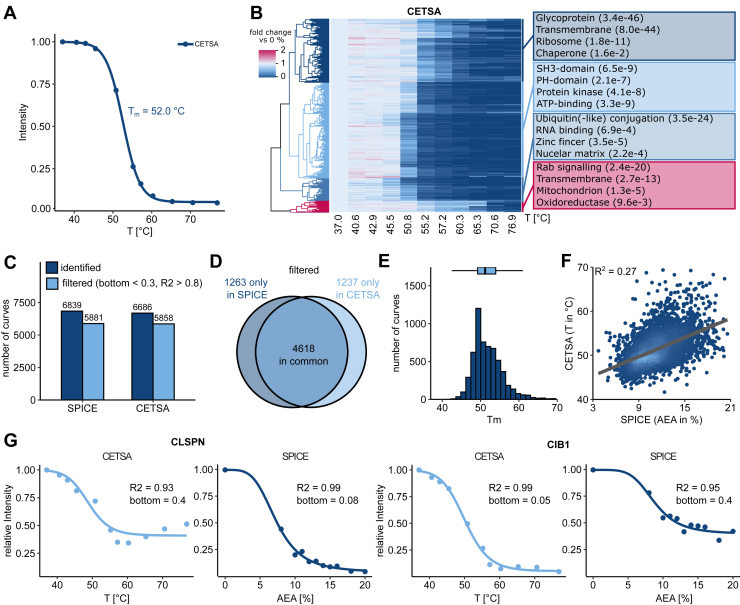
**SPICE generated curves are similar but complementary to CETSA generates curves**. *A*, median curves from CETSA experiments. *B*, heatmap of denaturation curves from SPICE experiments. Intensities for each protein are shown relative to the intensity at 0% AEA. Proteins are ordered by hierarchical clustering. *Boxes* on the right indicate functional clusters of annotations from multiple databases including GO, KEGG, and UniProt, that are enriched in the respective hierarchical cluster. All proteins with curves passing the quality criteria were used as background. *C*, number of proteins before and after filtering for high-quality curves in SPICE and CETSA. *D*, overlap of proteins in SPICE and CETSA after filtering. *E*, histogram of the T_m_-distribution in the CETSA experiment. *F*, correlation between denaturation points in SPICE and CETSA. Each dot represents one protein. *Lighter color* represents higher density of datapoints. *G*, denaturation curves from CLSPN and CIB1 in SPICE and CETSA. CETSA, cellular thermal shift assay; AEA, acetone:ethanol:acetic acid; SPICE, solvent proteome profiling in cells; GO, Gene Ontology; KEGG, Kyoto Encyclopedia of Genes and Genomes.

Having established that SPICE allows to detect cell-treatment specific stability changes, we sought to compare it with CETSA, which is the most commonly used label-free method to identify compound protein interactions and their resulting downstream effects on protein stability. We therefore generated denaturation curves in a CETSA experiment equivalent to the previously described SPICE experiment, using heat instead of organic solvents for denaturation (Supplemental Dataset S3). We detected an average melting temperature of 52 °C, consistent with previously described CETSA experiments (14, 15) (Fig. 3*A*). Clustering of CETSA curves and functional enrichment of the clusters allowed similar observations as in SPICE: DNA- and RNA-binding proteins are enriched in clusters of less stable proteins, while (mitochondrial) transmembrane proteins, glycoproteins, and proteins related to Rab-signaling cluster together in clusters of relatively high stability (Figs. 3 and Supplemental Fig. S2, *A*–*D*).

In comparison to the SPICE experiment, we observed a similar number of identified proteins and proteins with high-quality denaturation curves (Fig. 3*C*). The identified proteins mostly overlapped between both datasets (Fig. S2e), due to equal lysis and protein extraction steps after denaturation. However, for distinct subsets of proteins, only one of the methods allowed generation of a high-quality melting curve (Fig. 3*D*). SPICE and CETSA therefore complement each other in terms of the fraction of the proteome, for which a high-quality denaturation curve can be generated. Upon comparison of curve fit parameters for proteins with high-quality melting curves in both assays, slopes are steeper for CETSA curves (Supplemental Fig. S2*F*) and melting temperatures in CETSA seem to be more diverse than half-denaturation concentrations in SPICE (Fig. 3*E*). Denaturation points correlate weakly between both assays (Fig. 3*F*), while the bottom plateaus do not correlate (Supplemental Fig. S2*G*). Both observations help to explain the differences in proteins with high quality denaturation curves between both sets, since a subset of proteins seem to be differentially susceptible to denaturation by heat or solvents. Especially the incomplete denaturation of proteins like CLSPN and CIB1, which do not reach a sufficiently low bottom plateau despite a good curve fit in at least one of the assays (Fig. 3*G*), is not dependent on measurement depth or quantitative reproducibility, highlighting the complementarity of both assays.

### SPICE Enables Target Identification and Mechanism of Action Deconvolution in a Pooled Format

Recording and analyzing full melting curves of thousands of proteins is time-consuming and material-intense, often results in small effect sizes and allows curve-fitting only for proteins which follow a simplistic denaturation model (58). We established a compressed format of the workflow, to overcome those limitations and to facilitate systematic compound profiling with SPICE. Similar formats, which estimate the area under a denaturation curve have previously been developed for TPP (29) and adapted for SIPP (30). Soluble fractions from samples of the same denaturation curve are pooled and measured as a single “compressed” sample, which represents the area under the denaturation curve. Protein stabilization is detected by increased intensities in the pooled sample (Supplemental Fig. S3*A*). For compressed SPICE, we selected a range of AEA concentrations (8, 10, 12, 14, 16, 18, 20, and 22% (v/v) AEA) balancing previous observations, that narrower optimized ranges of denaturing conditions can lead to increased effect sizes (30, 59), with the risk of missing a curve shift that happens outside of the pooled range.

**Fig. 4:**
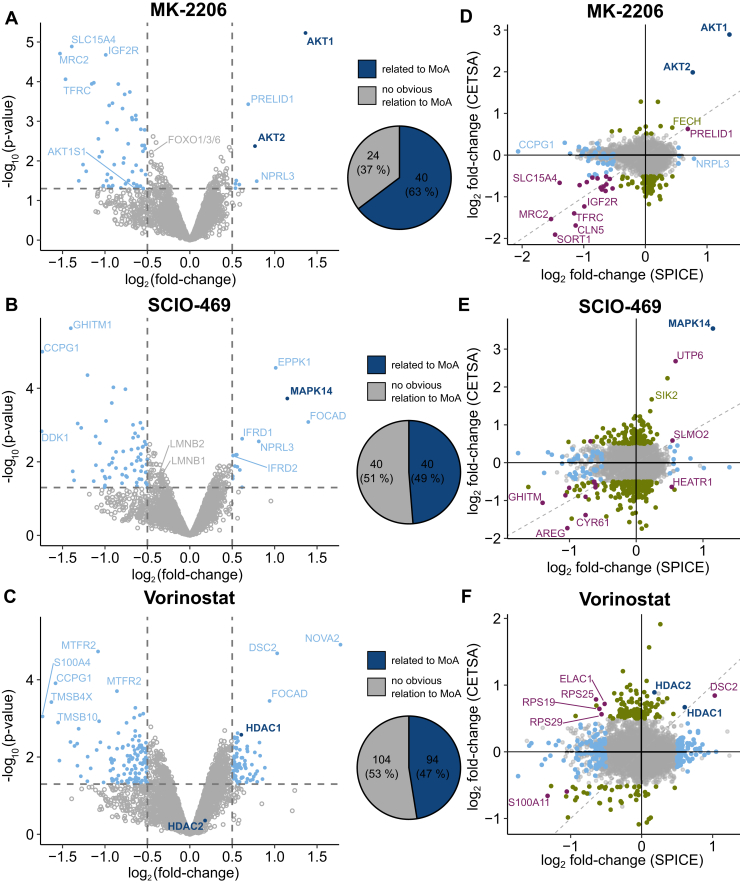
**SPICE in a compressed format allows detection of MoA-related protein stability changes**. *A–C*, HCT116 cells were treated with 25 μM MK-2206 (*B*), 100 μM SCIO-469 (*C*) or 25 μM Vorinostat (*D*) and analyzed by SPICE in a pooled format. Volcano plots (*left*) depict the fold change of protein intensities compared to the DMSO control against the respective statistical significance with proteins surpassing the cutoffs for significance (*p* value <0.05) or fold-change (>2) shown in *light blue*. Known protein targets of the respective compounds are shown in *dark blue*. The fraction of significantly changing proteins that are related to the compounds mechanism of action compared to all proteins above the cutoffs is shown as a pie chart. *D–F*, comparison of the stability changes observed in compressed SPICE experiments with stability changes of equivalent PISA experiments. Known direct targets of the used compounds are shown in *dark blue*. Proteins passing significance and fold-change cutoffs in only one experiment are colored *light blue* (SPICE) or *green* (CETSA). Proteins passing both cutoffs in both experiments are colored *purple*. CETSA, cellular thermal shift assay; PISA, proteome integral solubility alteration; MoA, mechanism of action; SPICE, solvent proteome profiling in cells; DMSO, dimethyl sulfoxide.

We applied compressed SPICE to analyze compounds that have recently been profiled by SIPP (30): The ATP-competitive inhibitor of p38-MAP-kinase MAPK14 SCIO-469 (60), the allosteric inhibitor of the kinases AKT1 and AKT2 MK-2206 (61) and the histone deacetylase inhibitor Vorinostat (62). We first replicated previous SIPP results (30) and identified the main targets of the three model compounds as stabilized in HCT116 lysates (Supplemental Fig. S3, *B*−*D* and Supplemental Datasets S4−S6). To determine whether SPICE can detect stabilization of direct targets and whether SPICE can be used to identify downstream effects related to the MoA, we conducted SPICE experiments with live HCT116 cells treated for 1 hour with 100 μM SCIO-469, 25 μM MK-2206, 25 μM Vorinostat, or a vehicle control. Four out of five targets identified by SIPP were significantly stabilized by the respective compound (Fig. 4, *A*−*C*, Supplemental Datasets S7−S9). In addition, we could functionally relate, on average, 53% of the significantly stabilized or destabilized proteins to the respective mechanisms of action (Supplemental Table S1) and confirmed these observations with functional enrichment analyses of those proteins.

For MK-2206, we detected eight stabilized proteins including AKT1, and AKT2, as well as 56 destabilized proteins (Fig. 4*A*). 63% of (de-)stabilized proteins can be directly linked to MK-2206’s MoA, and significantly enriched annotation terms belong to three functional clusters: (i) transmembrane and signaling domain containing proteins, (ii) lysosomal (membrane) proteins, and (iii) endosomal (membrane) proteins (Supplemental Fig. S4*A*). These enriched annotation clusters are consistent with known biological consequences of AKT inhibition and with the role of AKT2 for regulating lysosome related processes (63, 64). A few remarkable examples of destabilized proteins can be used to trace the effects of AKT inhibition in HCT116 cells: (i) destabilization of AKT1S1, a known AKT1 substrate and interactor (65), indicates decreased phosphorylation by reduced AKT1 activity; (ii) destabilization of IGF2R, a direct interactor of AKT kinases (66), and of TFRC, an interactor of AKT1 (67), indicate reduced interaction with AKT upon treatment; (iii) destabilization of the low-density lipoprotein receptor, a receptor upstream of AKT signaling (61), might be the result of feedback loop signaling and reduced phosphorylation; and (iv) destabilization of FOXO transcription factors which are downstream effectors of AKT signaling, potentially caused by altered phosphorylation or activity upon AKT inhibition (68).

For SCIO-469, we detected 13 significantly stabilized proteins including the target MAPK14 as well as 67 significantly destabilized proteins (Fig. 4*B*). Fifty percent of the (de-)stabilized proteins are functionally linked to the MoA of SCIO-469. They show significant enrichment for signal domain containing proteins (Supplemental Fig. S4*B*) and include proteins from multiple signaling pathways involving MAPK14, such as MAPK signaling, epidermal growth factor signaling, transforming growth factor signaling, and nerve growth factor signaling. Two direct interactors of MAPK14, LMNB1, and LMNB2, are also significantly destabilized only slightly inside the fold-change cutoffs. We assume the stability of all of these proteins, as well as TBC1D2B, which has been associated with the interaction network of MAPK14 (69), to be perturbed because of changes in phosphorylation or protein-protein interaction as a result of disturbed kinase signaling during MAPK14 inhibition.

For Vorinostat, we observed 93 stabilized and 125 destabilized proteins, including HDAC1, but interestingly not HDAC2 (Fig. 4*C*). We could link 47% of the significantly modulated proteins to Vorinostat’s MoA. Thirty-eight proteins related to transcription, eleven proteins associated with chromatin-binding complexes and 10 proteins that are known to experience a change in gene expression upon histone deacetylase (HDAC) inhibitor treatment were significantly changed in this SPICE experiment. Furthermore, six proteins that are involved in mammalian target of rapamycin (mTOR) signaling, which has been shown to be influenced by Vorinostat (70), are significantly changed in stability. Lastly, Vorinostat has been shown to mitigate the phenotype of Huntington’s disease in mice (71), and we found twelve proteins with significantly changed stability in SPICE experiments, that are associated with the respective Kyoto Encyclopedia of Genes and Genomes (KEGG) pathway (Supplemental Fig. S4*C*). In sum, these data show that compressed SPICE is capable of detecting direct protein targets, but further allows to identify proteins and functional categories, which are linked to the compound’s MoA.

### Compressed SPICE Is Complementary to PISA

To test how compressed SPICE compares to PISA, we conducted PISA experiments equivalent to our SPICE experiments, only differing in the denaturation step (Supplemental Datasets S10-S12). In addition, 5374 protein identifications are shared between both experiments and 1239 and 854 proteins were exclusively detected in SPICE or CETSA, respectively (Supplemental Fig. S5*A*). These numbers are in line with the denaturation curve experiments. All designated targets of the three compounds tested were also significantly stabilized in CETSA (Supplemental Fig. S5, *B–D*). Interestingly, functional enrichment of stability-modulated proteins only partially agreed with the respective results from SPICE: For MK-2206, we observed enrichment of lysosomal and endosomal proteins, as in the SPICE experiment (Supplemental Fig. S6*A*). Significantly changed proteins for SCIO-469 and Vorinostat showed mainly enrichment in ribosomal and, for SCIO-469, mitochondrial proteins (Supplemental Fig. S6, *B* and *C*). Both are not directly related to the compounds known mechanisms of action but might be the consequence of a general stress response, which seems to be readily detectable with CETSA. We observed limited correlation between the compound-induced stability changes of the two methods. Some proteins even exhibited stability changes in opposite directions in SPICE and CETSA (Fig. 4, *D–F*).

In the case of MK-2206, PRELID1 is the only protein besides the direct targets AKT1 and AKT2 with significant stabilization above the chosen cutoffs in both assays but has no known relation to Akt signaling. Here, 18 destabilized proteins are shared between both assays, most of which we could associate with MK-2206’s mode-of-action (Supplemental Table S1). Some proteins, like FECH, which is a known off-target of kinase inhibitors (72), are significantly changed in one assay, but narrowly miss the cutoff in the other assay. These proteins might be detected as hits in the other assay under slightly varied conditions. Other proteins, like NRPL3, which we could link to MK-2206’s mode-of-action (64), are strongly stabilized in SPICE but not changed in CETSA. We would consider proteins from both cases as relevant true hits, reflecting the complementarity of both assays. This is further supported by the observation that even though enrichment of lysosomal and endosomal proteins was observed in SPICE and CETSA, only five lysosomal/endosomal proteins were commonly changed in stability (SORT1, CLN5, CTSZ, TFRC, and SORL1) while all other proteins that drove the respective enrichments were unique to one of the two assays. Consistently, we observed only a small number of shared hits for SCIO-469 and Vorinostat in SPICE and CETSA, respectively. Notably, both compounds resulted in a single commonly stabilized protein, in addition to the respective main targets, which has not yet been associated with the respective compound. The combination of SPICE and CETSA could serve here as a validation of initially unexpected stability changes. Overall, the results across all three compounds demonstrate that the two assays provide complementary insights regarding stabilized proteins, which are potentially conceivable as direct targets, and particularly in detecting protein stability changes downstream of direct drug-target interactions, enabling a more comprehensive elucidation of the respective mechanisms of action.

### SPICE Detects Protein Targets of the Covalent Inhibitor Ibrutinib

**Fig. 5:**
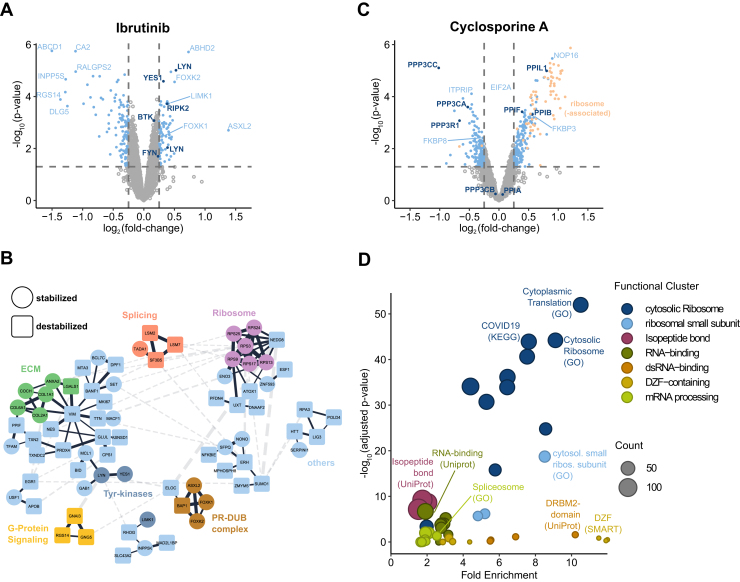
**SPICE can detect covalently bound targets and changes in compound-induced changes in protein-protein interactions**. K562 cells treated with 25 μM ibrutinib (n = 3) and HeLa cells treated with 120 μM cyclosporine A (n = 3) were analyzed by SPICE in a compressed format. *A* and *C*, volcano plots depict the fold change of protein intensities compared to the vehicle controls (n = 3) against the respective statistical significance with proteins surpassing the cutoffs for significance (*p* value <0.05) or fold-change (>2) shown in *light blue*. Known protein targets of the respective compounds are shown in *dark blue*. *B*, a STRING network was created with a minimal score of 0.4 for all proteins that lie above the significance and fold-change cutoffs for the ibrutinib experiment and was clustered by MCL clustering. All subnetworks with more than two members are shown. The colors of the nodes depict association with a known mechanism of action or common functional annotation, while the shape indicates the direction of the stability change. *D*, functional clusters of annotations that are significantly enriched cyclosporine A experiment are shown in a volcano plot, depicting the *p* value of the enrichment and the fold enrichment against all identified proteins in this experiment. Colors depict the association of each term with a functional cluster. Terms for functional clusters with an enrichment score >3 are shown. The source database for the respective term is given in *brackets*. MCL, Markov clustering algorithm; SPICE, solvent proteome profiling in cells.

To expand the variety of compound classes tested for SPICE, we profiled the covalent tyrosine kinase inhibitor ibrutinib, which targets Bruton’s tyrosine kinase (BTK) as well as multiple other protein kinases (73). K562 cells were treated with 25 μM ibrutinib for 1 hour and analyzed by SPICE with quantitative TMT-based proteomics. We detected significant destabilization of 115 proteins and stabilization of 71 proteins including several known targets of ibrutinib, such as YES, LYN, RIPK2, and FYN (Fig. 5*A* and Supplemental Dataset S13). BTK showed significant stabilization but with small effect size (fold change of 1.13). Systematic network analysis using STRING revealed a significantly interconnected network of 185 proteins (*p* = 9.4∗10^-14^) being physically and functionally related to the stabilized kinases. Clustering of this network using the Markov clustering algorithm (MCL) (74) resulted in multiple interconnected subnetworks related to G protein signaling, translation, splicing, and chromatin remodeling (Fig. 5*B* and Supplemental Table 2). The SIPP experiment in cell extract did not show significant stabilization of known ibrutinib targets under the tested conditions (Supplemental Fig. S7*A* and Supplemental Dataset S14). Conclusively, SPICE allowed detection of direct drug targets of a covalent drug as well as related downstream effectors, which were both elusive by SIPP.

### SPICE Detects Compound-Induced Modulation of Protein-Protein Interactions

To evaluate if SPICE can detect drug induced alterations of protein-protein interactions, we profiled the molecular glue cyclosporine A (CsA) with SIPP and SPICE. CsA binds directly to peptidyl-prolyl cis-trans isomerases (PPIases) (75) and induces their interactions with calcineurin (76). For SPICE, HeLa cells were treated with 120 μM CsA for 1 hour, and for SIPP, HeLa cell extracts were incubated with 120 μM CsA for 20 min prior to solvent-based denaturation. With SIPP, we observed CsA to stabilize 72 proteins and destabilize 26 proteins (Supplemental Fig. S7*B* and Supplemental Dataset S15). The stabilized proteins include three direct targets of CsA: PPIA, PPIB, and PPIF, with PPIB being the most significantly stabilized protein. Other proteins with changed stability include the calcineurin homolog TESC, the calmodulin dependent-kinase DCLK2 and four ribosome associated proteins, RPL35, RPLP2, RPL4, and EIF2A. The stability of calcineurin subunits was not changed. In the SPICE experiment, PPIF, PPIB, and PPIL1 were significantly stabilized among 216 other stabilized proteins (Fig. 5*C* and Supplemental Dataset S16). In contrast to the SIPP results, the three detected subunits of calcineurin (PPP3CA, PPP3CC, and PPP3R1) were significantly destabilized together with 183 other proteins.

To better understand the functional associations between significantly (de-)stabilized proteins in the SPICE experiment, we analyzed them for enrichment of functional annotations, followed by functional clustering of enriched terms. Proteins of the cytosolic ribosome, (ds)RNA-binding proteins and mRNA-processing proteins are significantly overrepresented (Fig. 5*D* and Supplemental Table S2). A network analysis of the same proteins using the STRING revealed a highly interconnected cluster of 403 functionally and physically interacting proteins (*p* = 10^-16^) including the PPIases and calcineurin subunits (Fig. S7*C*). Clustering of the network resulted in multiple subclusters. The largest cluster consists of 139 proteins including PPIB. Proteins in this cluster are mainly either part of the cytosolic ribosome or directly associated with it, like the stabilized EIF2A and the related and destabilized signaling kinase EIF2AK2. This is consistent with previous knowledge that CsA induces the unfolded protein response (UPR) (77, 78) and the integrated stress response (79), potentially by inhibiting PPIase-dependent protein folding in the endoplasmic reticulum (80). The UPR includes translational attenuation through phosphorylation of EIF2A and subsequent stalling of ribosomes (81), which in turn plausibly explains the stabilized EIF2A protein as well as stabilized ribosomal proteins. Furthermore, this cluster contains FKBP3 and FKBP8, which show significant but contrary changes in stability. Both are not known as direct interactors of CsA, despite structural similarity to peptidyl-prolyl cis-trans isomerases. They may nevertheless experience stability changes upon changed activity, stability, or phosphorylation of their common interactor calcineurin. The second biggest cluster consists of 62 proteins, including PPIL1, which are mostly RNA-binding. This cluster is highly connected to the cluster of ribosomal proteins and therefore also likely affected through the induced UPR. We almost exclusively observe stabilized proteins in these two clusters, which strengthens the assumption that a common biological effect like stalled ribosomes causes these stability changes. Several other smaller clusters contain a signaling kinase, which can be the reason for a stability change of associated proteins. In contrast to the ribosomal and RNA-related proteins, these kinases and other proteins related to intracellular signaling are mainly destabilized, hinting toward a common mechanism for destabilization, like phosphorylation changes.

### Combining SPICE and Full Proteome Profiling Allows to Analyze Degrader-Protein Interactions

Interpretation of cell-based experiments in a pooled format does not allow differentiating between protein abundance changes and protein stability changes, since both result in a change of protein amount being detected in the compressed sample. It is reasonable to assume negligible interference for short treatment durations and most compound classes, as protein abundances are regulated on a slower biological timescale. However, this interference cannot be ignored for compounds which modulate protein levels, either as part of their MoA (such as heterobifunctional and molecular glue degraders) or as rapid functional consequence of inhibition of their target. For the molecular glue CsA, we observed a strong and significant decrease in intensity of calcineurin subunits in the compressed SPICE experiment (Fig. 5*C*). However, since protein degradation, in contrast to protein synthesis, can occur fast enough, to cause the observed decrease of calcineurin subunits in CsA-treated SPICE samples within 1 hour of cell treatment, we could not exclude that these proteins are rapidly degraded and not biophysically destabilized.

**Fig. 6:**
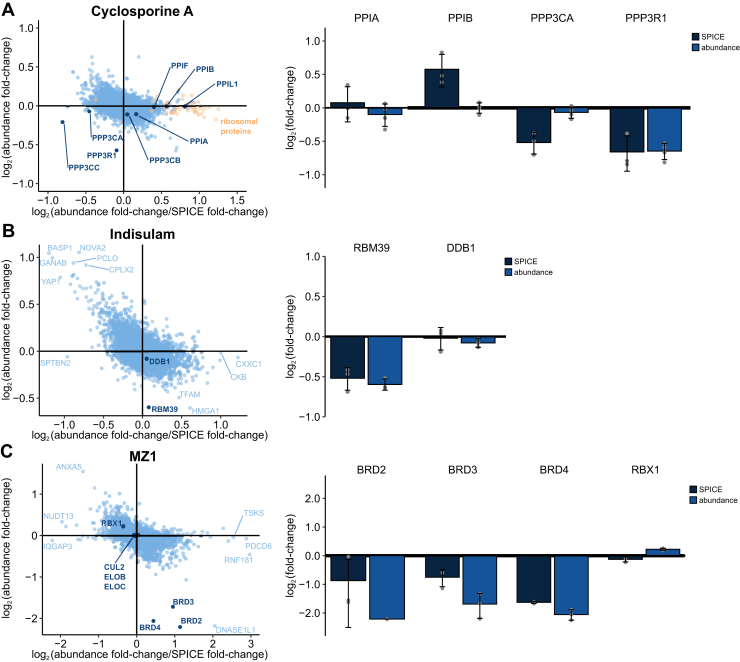
**SPICE can detect degrader-protein interactions**. HeLa cells treated with 120 μM Cyclosporine A (*A*) (n = 4), A549 cells treated with 20 μM Indisulam (*B*) and K562 cells treated with 20 μM MZ1 (*C*) (all n = 3) were analyzed by SPICE for changes in protein stability while aliquots of the same treated cells were analyzed for expression changes. Scatter plots (*left*) show the abundance fold change plotted against the fold change from the corresponding SPICE experiment divided by the respective abundance fold change. Known targets of the respective compounds are colored *dark blue*. Log_2_ fold changes for selected targets from both experiments are shown as bar plots (*right*). Error bars show the standard deviation. SPICE, solvent proteome profiling in cells.

To deconvolute changes in stability and protein level for calcineurin subunits and other relevant proteins, we performed global quantitative TMT-based proteome profiling of the same experimental conditions (HeLa cells, 120 μM CsA, 1 hour of treatment) and normalized the change in the SPICE experiment by dividing it by the change in the proteome profiling experiment. By doing so, we assume to cancel the proteome changes in the SPICE experiment and observe stability changes in the normalized SPICE results irrespective of protein level changes, akin to the previously described PISA-Express (82). As expected for such a short treatment time, only a small number of proteins displayed significant differential abundances (Supplemental Fig. S8*A*, Supplemental Dataset S17). For the ternary complex partners of the calcineurin:cyclophilin:CsA complex, we confirmed stabilization of various peptidyl-prolyl cis-trans isomerases, which did not show abundance changes (Fig. 6*A*). For the calcineurin complex, we observed minor degradation of three catalytic subunits of Calcineurin, which confirms the previous interpretation, that these proteins are destabilized by CsA treatment. In contrast, the regulatory subunit PPP3R1 is significantly degraded. Therefore, normalization of the decrease in intensity of PPP3R1 in SPICE to the decrease in intensity in the protein profiling experiment shows PPP3R1 not to be stability-modulated (Fig. 6*A* and Supplemental Fig. S8*A*). How destabilization and degradation are related mechanistically, warrants further studies.

To further investigate the necessity and usefulness for protein-level normalized SPICE, we analyzed the molecular glue Indisulam as well as the heterobifunctional proteolysis targeting chimera (PROTAC) MZ1 and its individual ligands, JQ1 and AHPC. We treated A549 cells for 1 hour with 20 μM Indisulam, a molecular glue known to induce degradation of RBM39 by recruiting the E3-ligase DCAF15 (83) and observed compound-induced reduction of RBM39 levels in SPICE and full proteome samples with similar effect sizes (Supplemental Fig. S8*B*, Supplemental Datasets S18 and 19). The normalized SPICE results clearly indicate that RBM39 is not stabilized or destabilized under the tested conditions but degraded (Fig. 6*B*). It is of note that Indisulam did stabilize CUL2 but none of the other complex members in SIPP experiments (Supplemental Fig. S8*C* and Supplemental Dataset S20).

To analyze the PROTAC MZ1 and its individual ligands, we treated K562 cells for 1 hour with 20 μM MZ1, which is known to induce degradation of bromodomain containing proteins BRD2, BRD3, and BRD4 (84). It binds these proteins through a JQ1-based moiety, while also recruiting the E3-ligase von Hippel-Lindau (VHL) through the VHL-binding moiety AHPC. To complement the MZ1 experiment, we included analyses of K562 cells treated for 1 hour with 100 μM of the BRD-binding moiety JQ1 or the VHL-binding moiety AHPC. As expected, treatment with MZ1 resulted in a significant reduction of BRD2, BRD3, and BRD4 in SPICE and full proteome samples, while VHL and associated proteins, such as the responsible E3 ligase RBX1 remained unchanged in both experiments (Supplemental Fig. S9*A* and Supplemental Datasets S21 and 22). When comparing normalized stability changes and abundance changes, it becomes clear that BRD2 and BRD3 are strongly stabilized, BRD4 to a lesser extent, and all three BRD proteins are degraded upon treatment (Fig. 6*C*). For JQ1, the SPICE experiment did not show stabilization of BRD2, BRD3, and BRD4, but their levels were reduced in the full proteome (Supplemental Fig. S9*B* and Supplemental Datasets S23 and 24). Consequently, the three proteins show normalized stabilization (Supplemental Fig. S9*C*). Other proteins that are stabilized in normalized SPICE are JMJD6, which is a direct interactor of BRD proteins (85), and 18 further proteins involved in the formation of the polymerase II elongation complex, including four of six subunits of the PFC1 complex (PAF1, CDC73, CTR9, and LEO1), and three polymerase II subunits (POLR2A/B/L). All these proteins are known to be recruited to DNA by BRD proteins (86), so the change in stability is likely caused by decreased recruitment to DNA as a consequence of inhibited BRD proteins. For the VHL-binder AHPC, the normalized stability experiment confirmed conclusions from the SPICE experiment. SPICE revealed small but not significant stabilization of VHL and its complex partner ELOB, while RBX1, also part of the VHL-E3-ligase complex, was slightly but significantly destabilized (Supplemental Fig. S9D and Supplemental Datasets S25 and 26). Full proteome profiling showed that RBX1 abundance was significantly increased, and consequently, the normalized SPICE results showed that RBX1 to be even more strongly destabilized (Supplemental Fig. S9*E*), possibly by perturbation of the VHL-complex upon binding of AHPC to VHL. Interestingly, the E3 ubiquitin-protein ligase RNF181 is the most stabilized protein after normalization of the SPICE effect, which fits to the known function of AHPC as an E3 ubiquitin-ligase binder. Notably, experiments with the heterobifunctional PROTAC MZ1 and its individual ligands highlight that SPICE in combination with full proteome profiling does not only allow deconvoluting stability and abundance changes but also allows characterizing the individual moieties of multifunctional compounds. Several observations, such as the stabilization of JMJD6 by JQ1 and RNF181 by AHPC, are consistent with the MZ1 results.

## Discussion

We developed SPICE as a novel biophysical proteomics method to study cellular drug-target interactions and downstream effects in a modification-free and proteome-wide manner without the need for *a priori* target or MoA hypotheses. To characterize the assay, we analyzed nine drugs and compounds from various mechanistic classes in three different cell lines (both adherent and suspension) using different formats of the assay. To evaluate broad applicability, the set of compounds and drugs comprised both classical chemical modalities, such as noncovalent orthosteric and allosteric enzyme inhibitors, natural compounds, targeted covalent inhibitors, and molecular glues or heterobifunctional degraders. To demonstrate the complementarity to SIPP and to CETSA-MS/TPP using living cells, we contrasted all SPICE experiments with the respective SIPP experiments and performed an in-depth comparison of SPICE to CETSA-MS, based on denaturation curves and a compressed area under the curve-approach (PISA) using three well characterized compounds.

Methodologically and conceptually, the SPICE assay shares most characteristics, applications, and formats with CETSA (15) and TPP (16), which is until now the only assay which allows studying proteome-wide target engagement and downstream effects of unmodified compounds in live cells by measuring the (altered) biophysical stability of endogenous proteins at their intrinsic abundance in their cellular environment. To detect an induced stability shift, both assays require that a significant proportion of the copies of each measured protein changes its stability (e.g. through high target occupancy). The conceptual similarity of the two assays is not only reflected by their possible applications (from screening via target deconvolution to biomarker discovery), but also by the plethora of conceivable formats, which match throughput, sensitivity, and readout requirements for these applications.

Comparison of SPICE and SIPP shows the well-known differences between lysate-based and cell-based TPP. SIPP is a biochemical assay and provides simpler results and straightforward interpretation regarding direct target-ligand binding. In contrast, SPICE as a cellular assay can detect drug targets *in cellulo* and provides a readout which is more complex and information-rich and inevitably demands further analysis and interpretation. For all tested compounds, we identified at least the designated target and detected downstream effects related to the compound’s MoA. It is well-known from TPP studies that secondary or downstream effects on protein stability arise from cellular effects that are caused by the inhibition or functional modulation of the respective targets. Altered biophysical stability has been shown to be conferred by e.g. changes in posttranslational modifications (16, 23, 87) or interactions with other proteins (21), nucleic acids (51), or metabolites (88) or other changes of a protein’s population. All these downstream events, which are a direct or indirect result of the perturbation, which happen at different biological timescales, and which can have opposite effects on protein stability, are integrated in the observed changes of protein stability.

Attributing the observed changes in SPICE experiments to biological processes, such as activation or deactivation of specific signaling pathways or stress responses, allowed us to associate many (often more than 50%) of the significantly modulated proteins to a compound’s MoA. We thus suggest that stability changes can serve as a “molecular marker” which highlights those proteins and pathways, which are worth being analyzed in more detail to generate robust hypotheses for follow-up studies. We think it is possible to draw a few conclusions on the direction of the secondary effect on protein stability from the SPICE experiments conducted in this study: Dephosphorylation, reduced metabolite concentrations, decreased recruitment or binding to DNA/chromatin and reduced protein-protein interactions lead to destabilization of a protein, while increased or induced protein-protein interactions, stabilization of protein complex partners, and increased interaction between DNA and histones lead to stabilization of involved proteins. With more SPICE, TPP and complementary functional proteomics studies analyzing abundance, posttranslational modifications or protein interactions, it will become clearer what the individual stability change of a protein means in a specific pharmacological context.

The key difference between TPP and SPICE is that SPICE requires applying a solvent mixture to live cells under otherwise physiological conditions, which likely gradually enters cells to denature and precipitate proteins, while TPP uses heat, which permeates cells almost instantaneously. We observed that SPICE denaturation is, by and large, comparable to heat-based protein denaturation in live cells. However, distinct differences remain, such as unique subsets of proteins which are not amenable to denaturation and only weakly correlating melting points, which together make up for valuable complementarity of these two in-cell denaturation approaches. When comparing SPICE and CETSA for drug target identification and mechanism-of-action profiling, we adapted a compressed format comparable with PISA, to enable fast profiling of compounds in live cells. Both approaches robustly identified the designated targets (and anecdotally known off-targets) with the same direction of stability change. Beyond that, the number of commonly stabilized or destabilized proteins is limited, but for each assay individually, the results are biologically meaningful and interpretable. We assume the complementarity of the results is a direct consequence of the melting characteristic of heat- and solvent-based protein denaturation in cells, and due to partly equivalent pooling ranges for the area under the curve compression.

For MK-2206, a small subset of overlapping and a large subset of nonoverlapping (de-)stabilized proteins commonly point toward modulation of lysosomal and endosomal processes, and thereby cross-validate the observations in either assay. For the other two test compounds, SCIO-469 and Vorinostat, we observed even smaller subsets of commonly (de-)stabilized proteins, and much more pronounced and divergent functional differences among the complementarily (de-)stabilized proteins. Accordingly, it appears as if the complementarity of temperature and solvent proteome profiling recently observed in experiments in cell extracts can also be observed in cell-based experiments, where the differences in stability-modulated proteins between both assays are even more pronounced. Together, this will help to resolve more drug targets and downstream proteins with altered biophysical stabilities than each assay alone and increases the confidence in the commonly observed effects, as it has been exemplified for other proteome-wide and label-free assays (25).

We anticipate that the simple scalability of solvent pipetting will be advantageous for increasing the throughput of SPICE. Together with recent and future advances in deep and high-throughput proteomics approaches using latest-generation LC-MS/MS systems and AI-based peptide and protein identification approaches (89), this will open the opportunity of profiling large numbers of compounds routinely using SPICE, for instance, for fast cell-based confirmation of hits identified by high throughput screening of compound libraries.

Conclusively, we show that SPICE allows studying drug target engagement and a drug’s MoA in live cells for various compound classes and in different formats. We anticipate that SPICE is broadly applicable, simply scalable and complements existing pharmacological and biophysical proteomics assays.

## Data Availability

The mass spectrometry proteomics data have been deposited to the ProteomeXchange Consortium via the PRIDE (90) partner repository with the dataset identifier PXD060621. reviewer_pxd060621@ebi.ac.uk

## Supplemental Data

This article contains supplemental data.

## Conflict of Interest

H. H. is cofounder and shareholder of OmicScouts GmbH. A. B., C. C., D. S., G. H., G. K., H. H., M. H. and S. K. M. and are employees of OmicScouts GmbH. D.S. and H. H. are the inventors of the SPICE technology (patent pending). The other authors declare no competing interests.
