# Supplementary material for: Proteome-Wide Monitoring of Drug Action in Living Cells Using a Novel Label-Free Solvent-Based Shift Assay
Source: Mol Cell Proteomics. 2025 Nov 5;24(12):101444. doi: 10.1016/j.mcpro.2025.101444 (PMC12802111; doi:10.1016/j.mcpro.2025.101444)
Supplement: Supplementary Dataset S26 [file mmc27.pdf]

Proteome-wide Monitoring of Drug Action in Living Cells using a Novel Label-free Solvent-based Shift Assay

Dominik Steinbrunn[1,2], Catalina Cepeleaga[1], Alexander Betz[1], Gözde Kibar[1], Melanie Holzner[1], Stefan K. Maier[1], Christin Zasada[1], Götz Hagemann[1], Stephan A. Sieber[2], Hannes Hahne[1]

[1]OmicScouts GmbH, Lise-Meitner-Straße 30, 85354 Freising, Germany

[2]TUM School of Natural Sciences, Department of Bioscience, Technical University of Munich, Center for Functional Protein Assemblies (CPA), 85748 Garching bei München, Germany

Supplemental Figures

Fig S1: Solvent Proteome Profiling In Cells (SPICE) is distinct from Solvent Proteome Profiling (SPP).

Fig S2: CETSA denaturation curves and comparison to SPICE.

Fig S3: SIPP for compounds used in SPICE experiments.

Fig S4: Functional enrichment of proteins with significant stability changes in compressed SPICE experiments.

Fig S5: PISA for comparison with compressed SPICE.

Fig S6: Functional enrichment of proteins with significant stability changes in PISA experiments.

Fig. S7: SIPP and SPICE for PPI modulators and covalent inhibitors.

Fig S8: Stability and abundance changes induced by Cyclosporine A and Indisulam.

Fig S9: Stability and abundance changes induced by AHPC, JQ1 and MZ1.

Supplemental Tables

Table S1: Proteins with MoA-related stability changes in compressed SPICE with non-covalent inhibitors.

Table S2: Proteins with MoA-related stability changes in compressed SPICE with Ibrutinib and Cyclosporine A.

Supplementary Figures

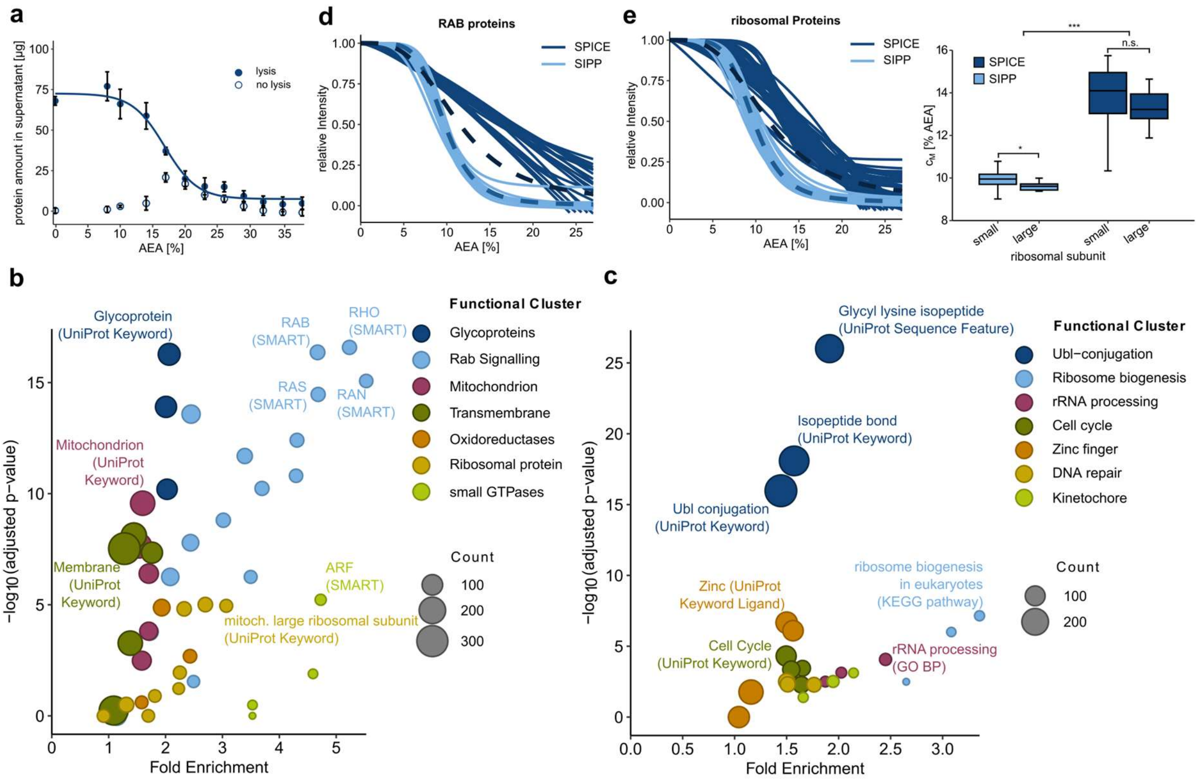

Figure S1 | Solvent Proteome Profiling In Cells (SPICE) is distinct from Solvent Proteome Profiling (SPP). a. Cells were subjected to the SPICE workflow (points) or to a modified SPICE workflow without specific lysis before centrifugation (circles). Protein amount of the soluble fractions was determined by a BCA assay and is plotted against the respective AEA concentrations. **b, c.** Functional clusters of annotations that are significantly enriched in the hierarchical SPICE denaturation curve clusters for late (**b**) and early (**c**) denaturing proteins are shown in a volcano plot, depicting the p-value of the enrichment and the fold enrichment against all identified proteins in this experiment. Colors depict the association of each term with a functional cluster. Terms for functional clusters with an Enrichment Score > 3 are shown. The most significant terms for each functional cluster are labelled. The source database for the respective term is given in brackets. **d, e.** Example curves of all Rab proteins (**d**) and all ribosomal proteins (**e**) identified in the SPICE and the SIPP experiment. Median curves of the respective experiments are shown as dashed lines. C_M-values derived from the denaturation curves of ribosomal proteins shown as a boxplot, separated by experiment and ribosomal subunit.

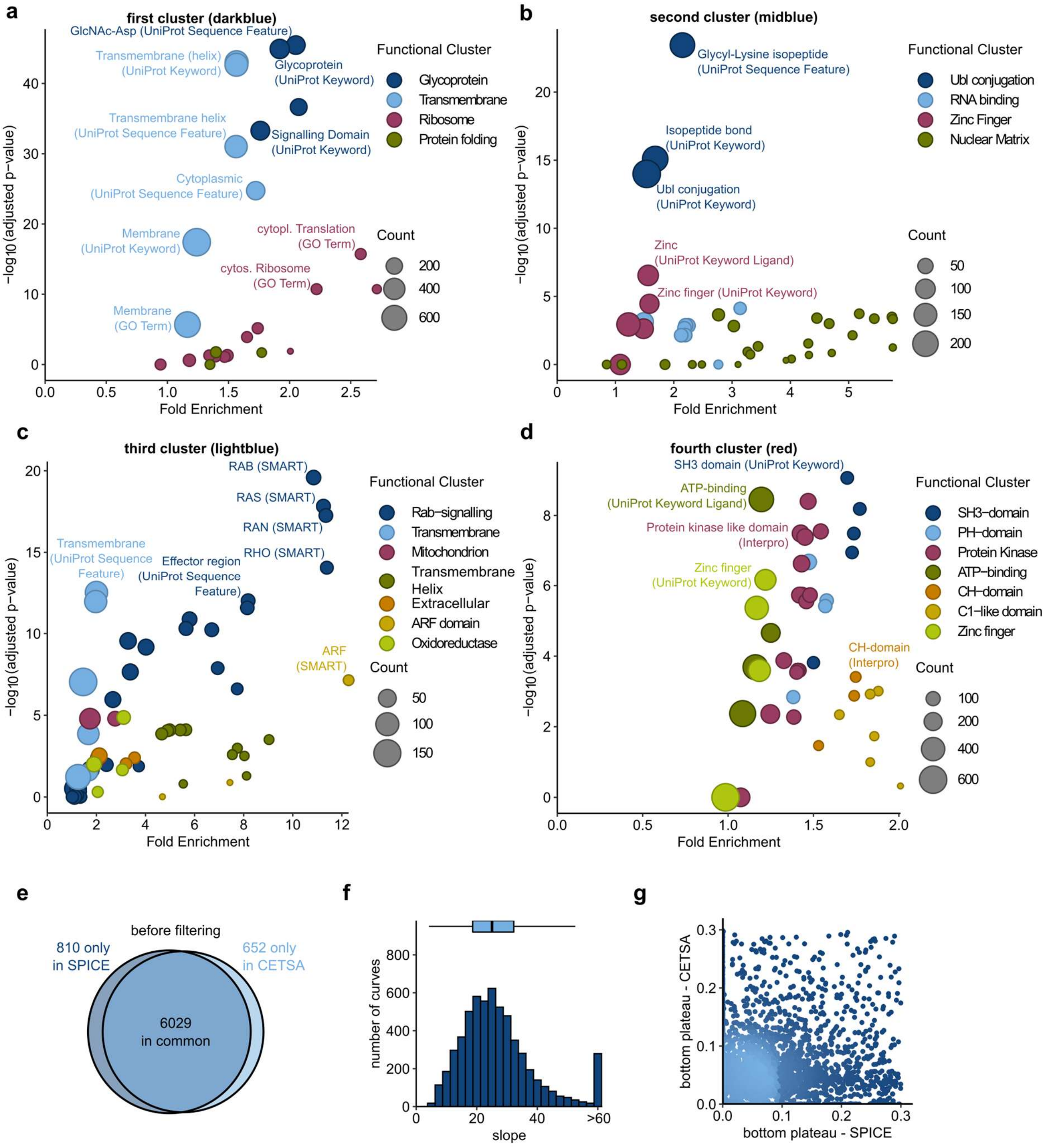

Figure S2 | CETSA denaturation curves and comparison to SPICE. a-d. Functional clusters of annotations that are significantly enriched in the hierarchical CETSA denaturation curve clusters are shown in volcano plots, depicting the p-value of the enrichment and the fold enrichment against all identified proteins in this experiment. Colors depict the association of each term with a functional cluster. Terms for functional clusters with an Enrichment Score > 3 are shown. The source database for the respective term is given in brackets. **e.** Overlap of proteins in SPICE and CETSA before filtering. **f.** Histogram for the distribution of slopes from CETSA denaturation curves. **g.** Comparison of the curve fit bottom plateaus from spice and CETSA after filtering. Each dot represents a protein. Lighter color represents higher density of datapoints.

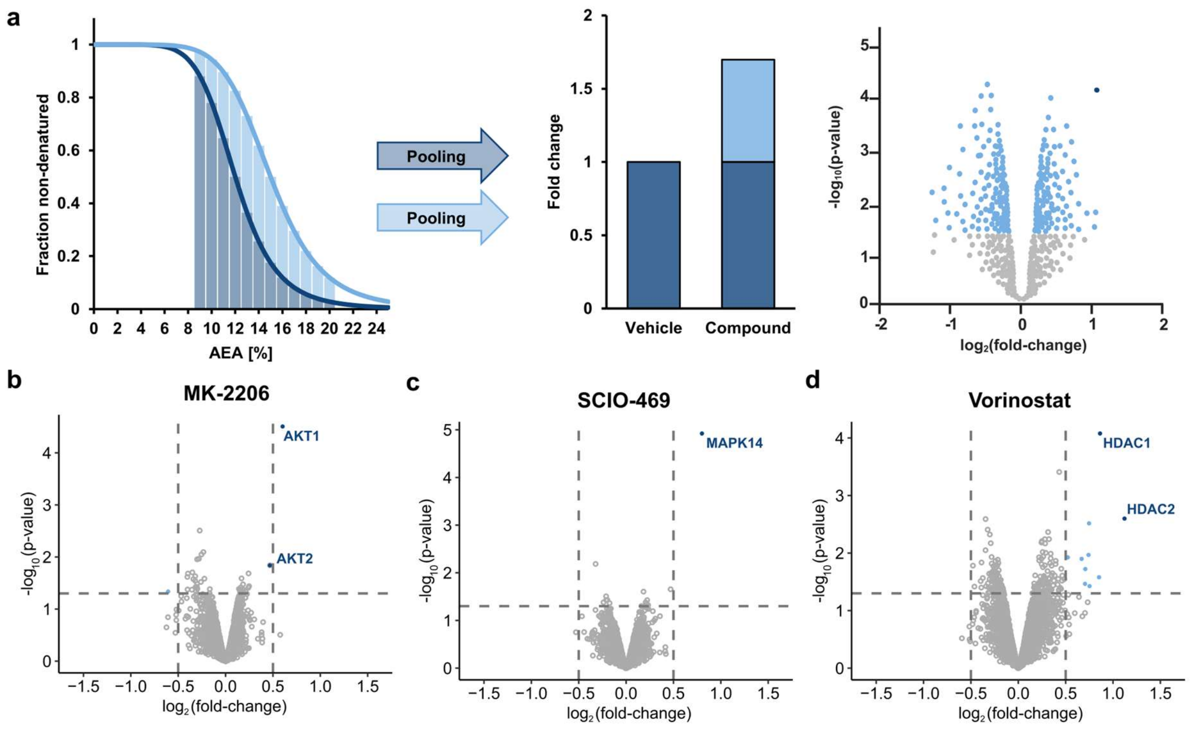

Figure S3 | SIPP for compounds used in SPICE experiments. a. HCT116 lysate treated with 25 µM MK-2206, 100 µM SCIO-469 or 25 µM Vorinostat were analyzed by SIPP (all n=3). Volcano plots depict the fold change of protein intensities compared to the DMSO controls (n=3) against the respective statistical significance. Proteins surpassing the cut-offs for significance (p-value < 0.05) or fold-change (> 2) are shown in light blue. Known protein targets of the respective compounds are shown in dark blue.

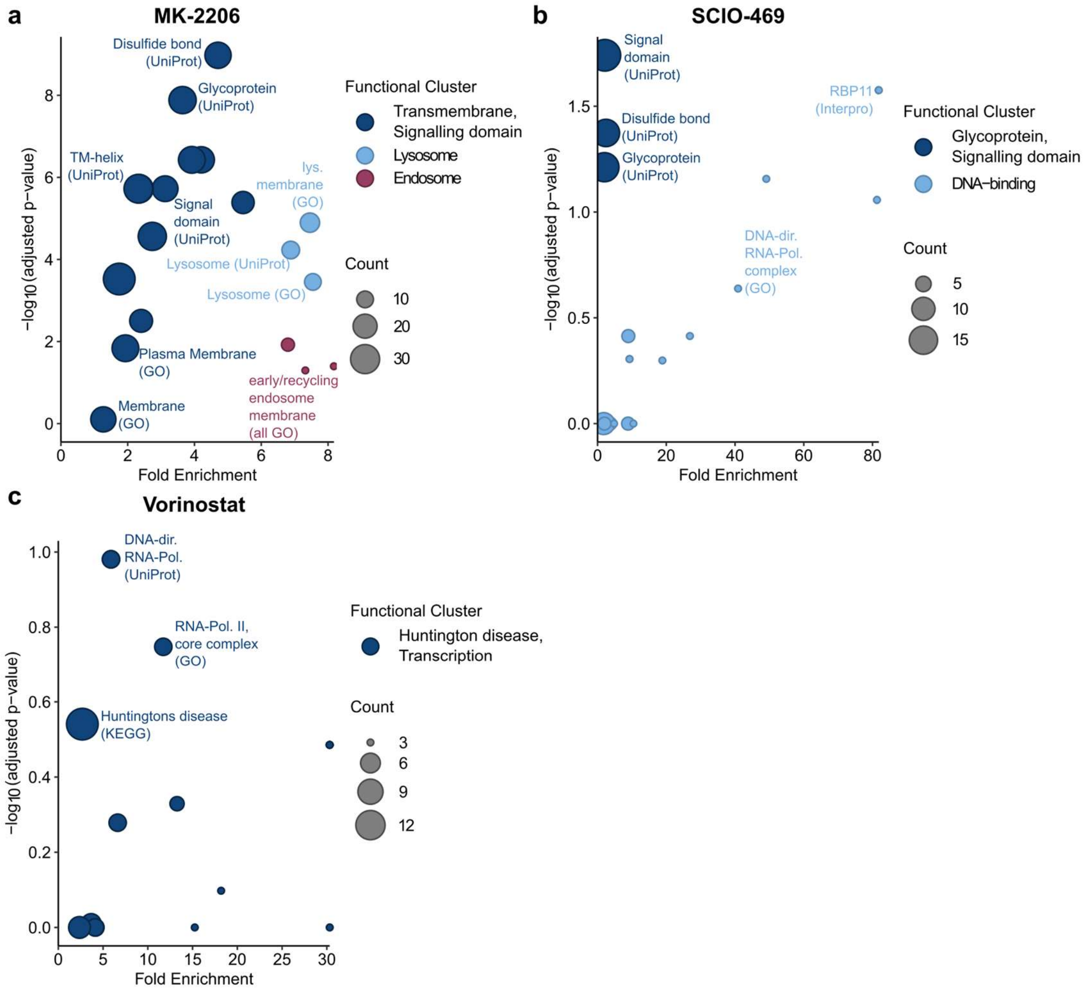

Figure S4 | Functional enrichment of proteins with significant stability changes in compressed SPICE experiments. a-c. Proteins surpassing the cut-offs for fold-change and significance in compressed SPICE experiments were analyzed for enrichment of annotations from multiple databases, followed by functional clustering of enriched terms. Volcano plots depict the p-value of the enrichment and the fold enrichment against all identified proteins in this experiment. Colors depict association with a functional cluster. Terms for functional clusters with an Enrichment Score > 3 are shown. The most significant terms for each functional cluster are labelled. The source database for the respective term is given in brackets.

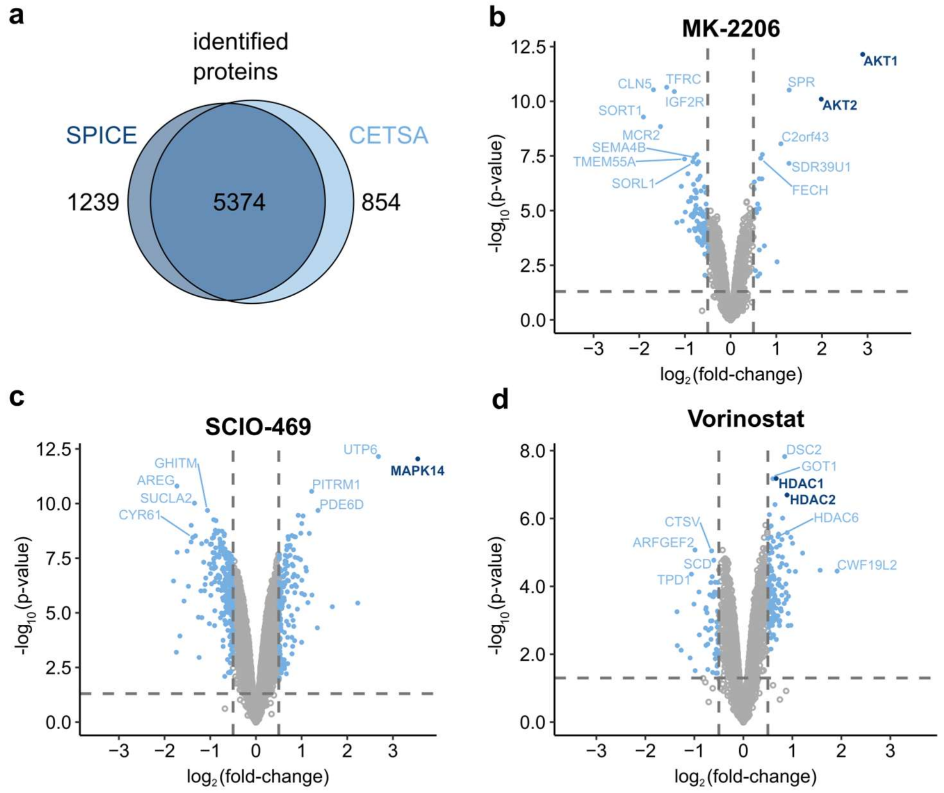

Figure S5 | PISA for comparison with compressed SPICE. HCT116 cells were treated with 25 µM MK-2206 (b), 100 µM SCIO-469 (c) or 25 µM Vorinostat (d) and analyzed by PISA. **a.** Overlap between proteins identified in compressed SPICE or PISA respectively. **b-c.** Volcano plots depict the fold change of protein intensities compared to the DMSO control against the respective statistical significance with proteins surpassing the cut-offs for significance (p-value < 0.05) or fold-change (> 2) shown in light blue. Known protein targets of the respective compounds are shown in dark blue.

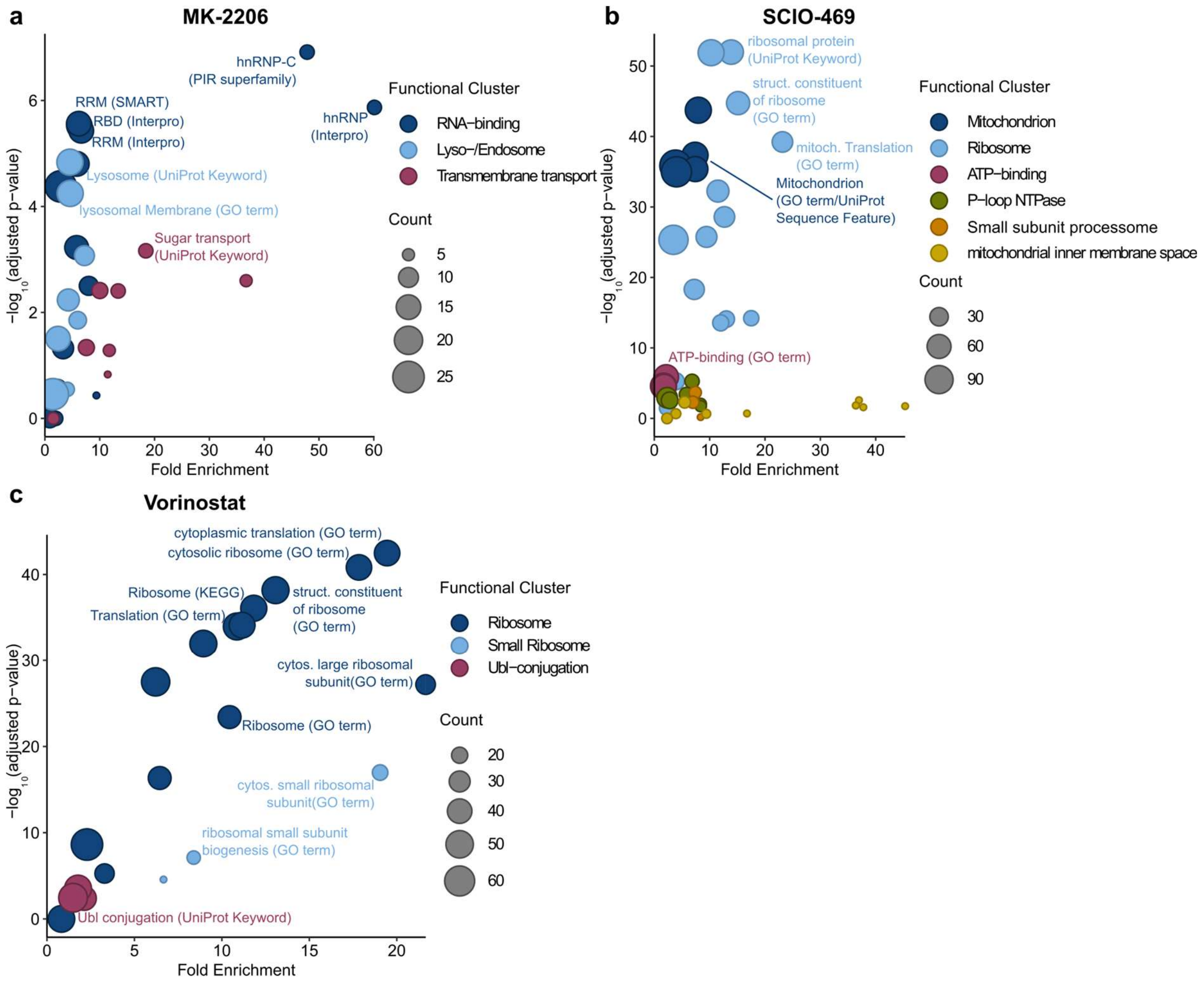

Figure S6 | Functional enrichment of proteins with significant stability changes in PISA experiments. a-c. Proteins surpassing the cut-offs for fold-change and significance in PISA experiments were analyzed for enrichment of annotations from multiple databases, followed by functional clustering of enriched terms. Volcano plots depict the p-value of the enrichment and the fold enrichment against all identified proteins in this experiment. Colors depict association with a functional cluster. Terms for functional clusters with an Enrichment Score > 3 are shown. The most significant terms for each functional cluster are labelled. The source database for the respective term is given in brackets.

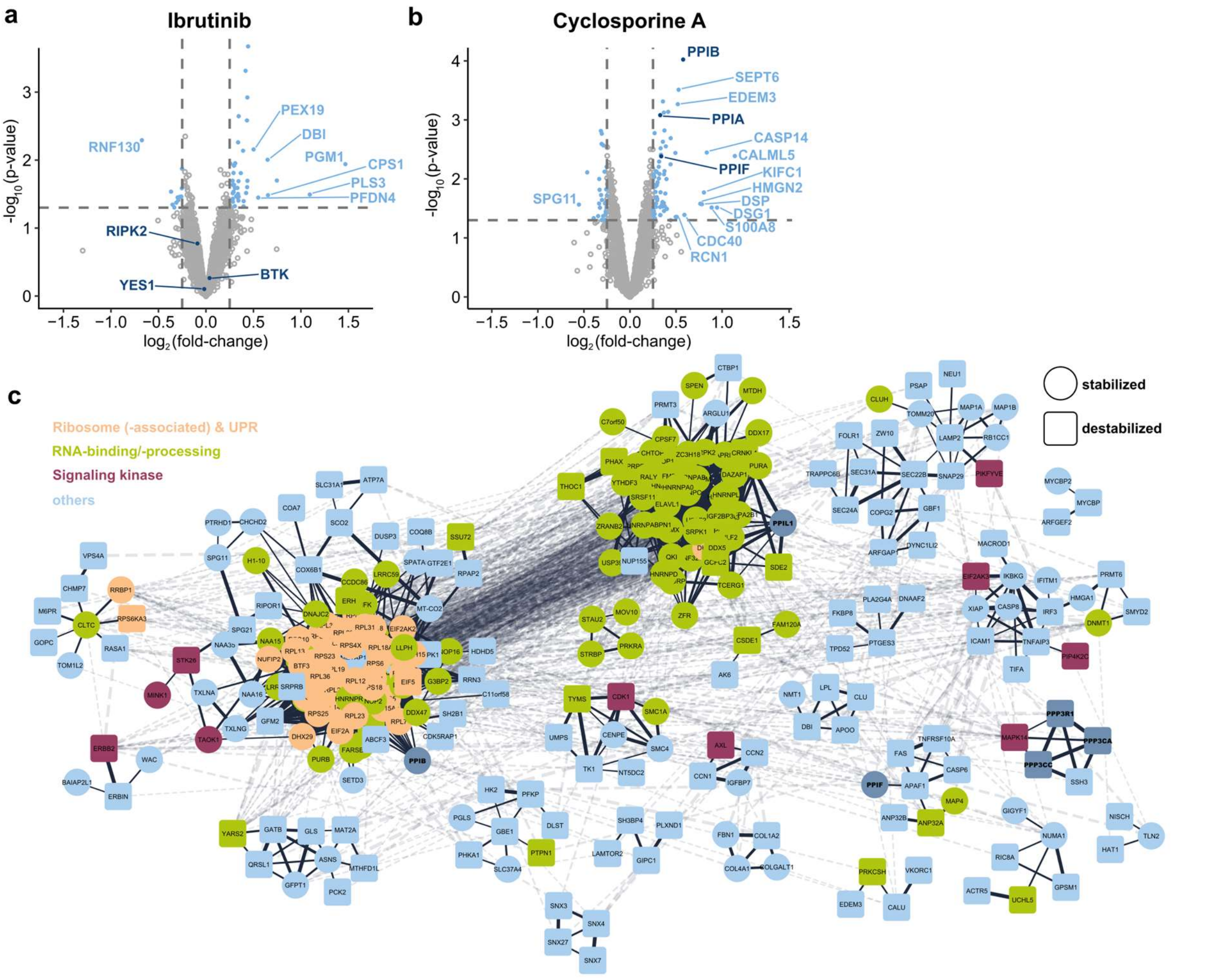

Figure S7 | SIPP and SPICE for PPI modulators and covalent inhibitors. K562 lysate was treated with 25 µM ibrutinib (**a**) and HeLa lysate was treated with 120 µM Cyclosporine A (**b**) before analysis by SIPP (n=3). Volcano plots depict the fold change of protein intensities compared to the vehicle controls (n=3) against the respective statistical significance with proteins surpassing the cut-offs for significance (p-value < 0.05) or fold-change ($\log_2$(fc) > ± 0.25) are shown in light blue. Known protein targets of the respective compounds are shown in dark blue. **c.** A STRING network was created with a minimal score of 0.4 for all proteins that lie above the significance and fold-change cut-offs for the Cyclosporine A SPICE experiment and was clustered by MCL clustering. All subnetworks with more than two members are shown. The colors of the nodes depict association with a known mechanism of action or common functional annotation, while the shape indicates the direction of the stability change.

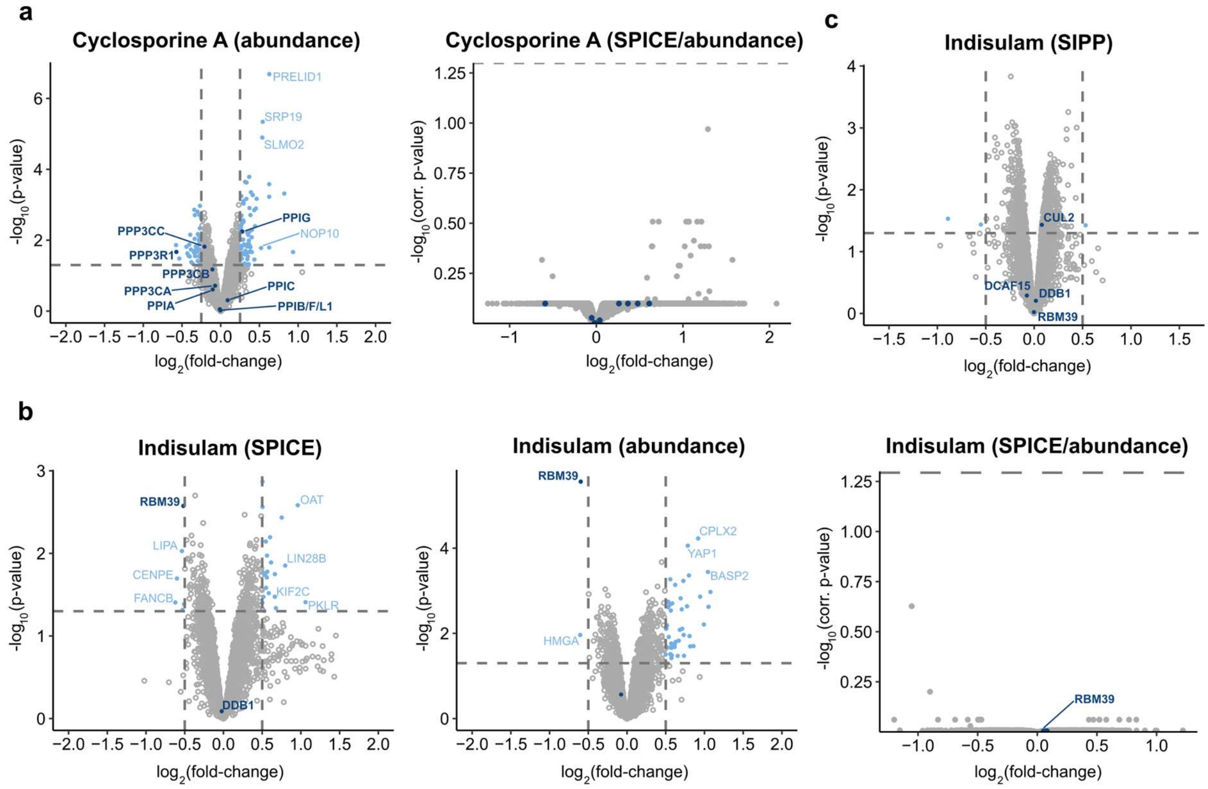

Figure S8 | Stability and abundance changes induced by Cyclosporine A and Indisulam. HeLa cells treated with 120 µM Cyclosporine A (**a**) and A549 cells treated with 20 µM Indisulam were analyzed for protein stability changes by SPICE and for abundance changes (all n=3). Volcano plots depict the fold change of protein intensities compared to the DMSO controls (n=3) against the respective statistical significance with proteins surpassing the cut-offs for significance (p-value < 0.05) or fold-change (> 2) are shown in light blue. Known protein targets of the respective compounds are shown in dark blue. Results from SPICE are shown left and results from analysis of abundance changes are shown in the middle. A volcano depicting the contrast of both fold changes for each protein is shown on the right. (**c**) K562 lysates were treated with 20 µM Indisulam and analyzed by SIPP (n=3).

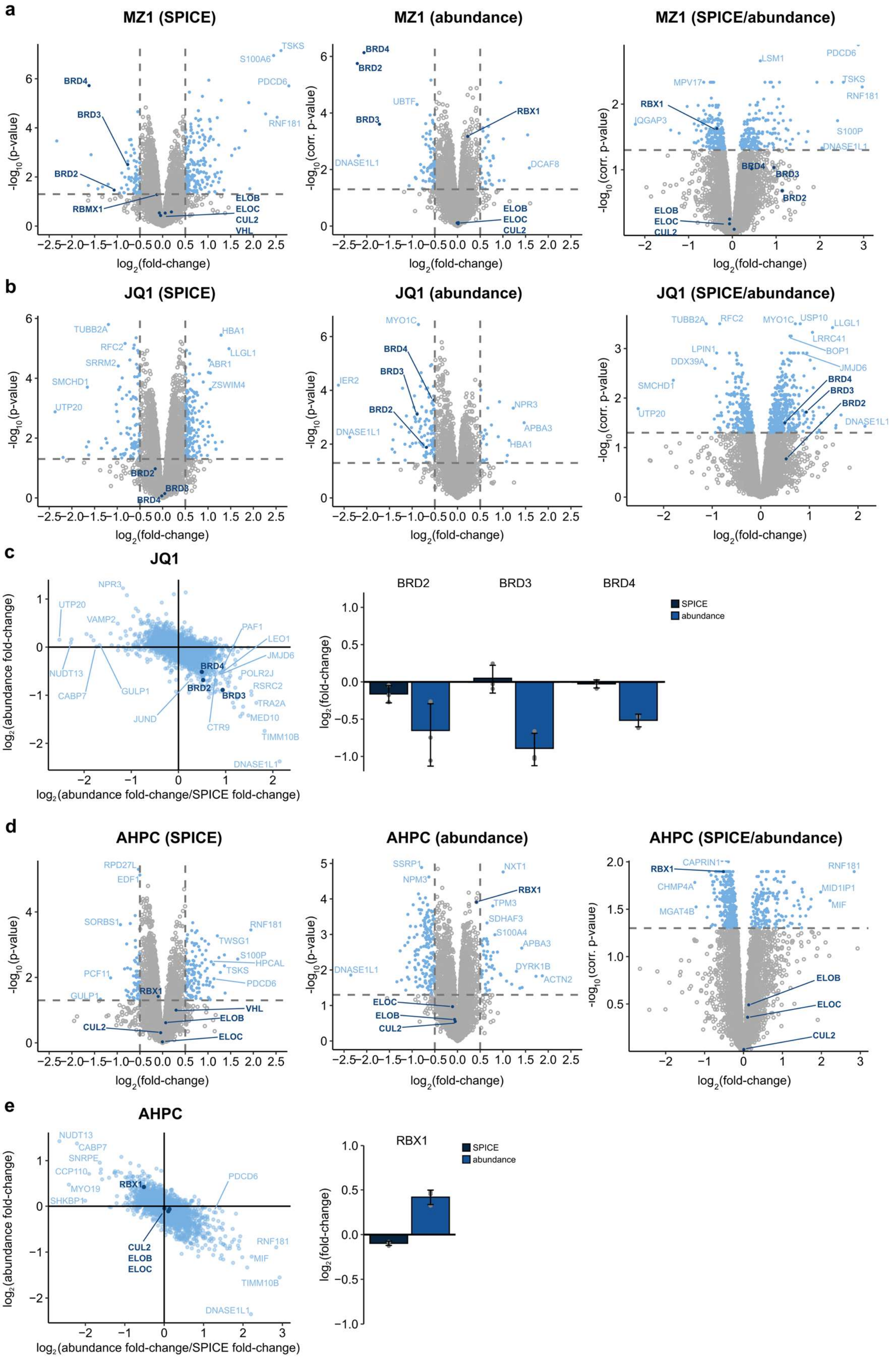
a
MZ1 (SPICE)
MZ1 (abundance)
MZ1 (SPICE/abundance)
-log10(p-value)
-log10(corr. p-value)
log2(fold-change)
TSKS
S100A6
PDCD6
RNF181
BRD4
BRD3
BRD2
RBMX1
ELOB
ELOC
CUL2
VHL
UBTF
DNASE1L1
RBX1
DCAF8
LSM1
MPV17
IQGAP3
S100P
b
JQ1 (SPICE)
JQ1 (abundance)
JQ1 (SPICE/abundance)
TUBB2A
RFC2
SRRM2
SMCHD1
UTP20
HBA1
LLGL1
ABR1
ZSWIM4
MYO1C
IER2
NPR3
APBA3
USP10
LRRC41
BOP1
JMJD6
LPIN1
DDX39A
c
JQ1
log2(abundance fold-change)
log2(abundance fold-change/SPICE fold-change)
NPR3
VAMP2
NUDT13
CABP7
GULP1
JUND
PAF1
LEO1
POLR2J
RSRC2
TRA2A
MED10
TIMM10B
CTR9
log2(fold-change)
SPICE
abundance
d
AHPC (SPICE)
AHPC (abundance)
AHPC (SPICE/abundance)
RPD27L
EDF1
SORBS1
PCF11
GULP1
TWSG1
HPCAL
SSRP1
NPM3
NXT1
TPM3
SDHAF3
S100A4
DYRK1B
ACTN2
CAPRIN1
CHMP4A
MGAT4B
MID1IP1
MIF
e
AHPC
NUDT13
CABP7
SNRPE
CCP110
MYO19
SHKBP1
RBX1

Figure S9 | Stability and abundance changes induced by AHPC, JQ1 and MZ1. K562 cells treated with 20 µM MZ1 (**a**), 100 µM JQ1 (**b**, **c**) or 100 µM AHPC (**d**, **e**) (all n=3) were analyzed by SPICE for changes in protein stability while aliquots of the same cells were analyzed for expression changes. Scatter plots show the abundance fold change plotted against the fold change from the corresponding SPICE experiment divided by the respective abundance fold change. Known targets of the respective compounds are colored dark blue. Log_2 fold changes for selected targets from both experiments are shown as bar plots (right). Error bars show the standard deviation. Volcano plots depict the fold change of protein intensities compared to the DMSO controls (n=3) against the respective statistical significance with proteins surpassing the cut-offs for significance (p-value < 0.05) or log_2 fold-change (> 0.5) are shown in light blue. Known protein targets of the respective compounds are shown in dark blue. Results from SPICE are shown left and results from analysis of abundance changes are shown in the middle. A volcano depicting the contrast of both fold changes for each protein is shown on the right.

Compound	Biological Process	Proteins
**MK-2206** (Fig. 4a)	**Target**	AKT1, AKT2
	**Direct interactor of target**	AKT1S1, IGF2R, TFRC
	**Endo-/lysosome**	ABCB6, BLOC1S3, CLN5, CPVL, CTSZ, ITM2C, NPRL3, SLC12A9, SLC15A4, SLC17A5, SORL1, SORT1, STX7, TPCN1, VTI1B, WASH1/2P/3P
	**Akt-signalling**	AKT1S1, BBS7, C3orf58, CD40, CNPY2, COL6A3, GREB1L, HES1, IGF2R, ITGA5, LDLR, LPIN3, NPRL3, PPP1R14B, RALGAPA1, S100A4, S100A6, STARD3, TFRC, TMSB10, TMSB4X, TNFRSF12A, TPD52
	**Insulin signalling**	LDLR, TFRC
**SCIO-469** (Fig. 4b)	**Target**	MAPK14
	**MAPK signalling**	APLP2, AREG, CCPG1, CD40, CYR61, DKK1, ENTPD4, FAM60A, GCSH, HEATR1, IFRD1, IFRD2, JAG1, JUND, LAP3, MT2A, OSMR, RNF126, S100A6, S100A11, SAR1A, SCD4, SLC7A1, TIMP1, TMSB10, TRIM26
	**EGFR signalling**	AREG, EPPK1, TRAF4, ZBTB7A
	**TGF signalling**	HES1, JAG1, KLF10, SAR1A
	**NGF signalling**	IFRD1, IFRD2, GHITM
	**growth factor**	MDK
**Vorinostat** (Fig. 4c)	**Target**	HDAC1
	**transcription**	ABHD14B, ARF4, ASF1B, ATF2, ATN1, ATXN3, BTF3L4, CCPG1, CD40, CPNE1, CUX1, DCAF7, DKK1, DNAJC17, FUBP1, GSDMD, HES1, HOXB8, INTS9, JUNB, JUND, NCK2, NFIC, NRBF2, PHF10, PHF5A, POLR2H, POLR2I, POLR2J, SMYD2, SNF8, SQSTM1, TFAM, TIMELESS, TOX4, USF2, VEZF1, ZBTB7A
	**mTOR signalling**	AKT1S1, ATP6V1G1, DAP, MLST8, RHEB, RRAGC
	**chromatin(-associated) complex**	ANKRD10, CCDC101, ERH, FAM111B, H2AFY2, ORC4, RPA2, SIGMAR1, STAG2, TBC1D2, ZMYM3
	**expression changed by HDAC inhibition**	CDA, CLDN3, CLDN7, CST6, FLYWCH2, LGALS1, RGS10, RRM2B, S100A4, SOD2

Table S1 | Proteins with MoA-related stability changes in compressed SPICE with non-covalent inhibitors. Proteins which passed significance and fold-change cut-offs in compressed SPICE with MK-2206, SCIO-469 and Vorinostat were grouped into categories related to the respectively known mechanisms of actions.

Compound	Biological Process	Proteins
**Ibrutinib** (Fig. 5a)	**Target (other Tyr-kinases)**	LYN, RIPK2, YES1, (TTN, LIMK1)
	**secreted proteins**	ANXA2, APOB, CDC37L1, CFAP54, COCH, COL1A1, COL2A1, COL6A1, DNAAF2, ICAM4, IGFBP4, LGALS1, LYZ, MAGED2, MDK, PRDX4, TNFRSF1B, TTN
	**innate immune response**	HOOK2, LYN, NONO, PPP1R14B, PYCARD, RIPK2, SEC14L1, SFPQ, YES1, ZCCHC3
**Cyclosporine A** (Fig. 5b)	**Target**	PPIB, PPIF, PPIL, PPP3CA, PPP3CC, PPP3R1
	**ribosome(-associated) & UPR**	34 RPLs, 27 RPSs, EHBP1L1, EIF2A, EIF2AK2, DHX29, FUBP3, NUFIP2, RRBP1, ZC3H15
	**RNA-binding/-processing**	17 HNRNPs, CAPRIN1, CARHSP1, CHTOP, CLUH, CPSF7, CRNKL1, CSDE1, DAZAP1, DDX17, DDX47, DDX5, DHX9, DIMT1, EIF2A, FAM120A, FASTKD5, FMR1, G3BP2, GCFC2, IGF2BP3, ILF3, MOV10, NIFK, NOP2, NUFIP2, PABPN1, PHAX, PNN, PRKRA, PRPF4, QKI, RALY, RBMX, RBMXL1, SDE2, SPEN, SRPK1, SRPK2, SRSF11, SSU72, STAU2, STRBP, THOC1, U2AF2, U2SURP, UPF3B, USP39, YTHDF3, ZFR, ZNF326, ZRANB2
	**Kinase**	AK6, AXL, CAMK1D, CDK1, CDK16, DCLK2, EIF2AK2, EIF2AK3, ERBB2, HK2, IKBKG, MAPK14, MINK1, PCK2, PFKP, PIKFYVE, PIP4K2C, PRKCSH, PRKRA, RPS6KA3, SRPK1, SRPK2, STK26, TAOK1, TK1
	**Ca-signaling**	ADCY9, ASPH, CAMKD1, ERBB2, ITPRIP, PPIF, PHKA1, PPP3CA, PPP3CC, PPP3R1

Table S2 | Proteins with MoA-related stability changes in compressed SPICE with Ibrutinib and Cyclosporine A. Proteins which passed significance and fold-change cut-offs in compressed SPICE with Ibrutinib and Cyclosporine A were grouped into categories related to the respectively known mechanisms of actions.
